# Supplementary material for: A Quantum Chemical Deep-Dive into the π-π Interactions of 3-Methylindole and Its Halogenated Derivatives—Towards an Improved Ligand Design and Tryptophan Stacking
Source: Pharmaceuticals (Basel). 2022 Jul 28;15(8):935. doi: 10.3390/ph15080935 (PMC9414876; doi:10.3390/ph15080935)
Supplement: Supplementary file 1 [file pharmaceuticals-15-00935-s001.zip › pharmaceuticals-1811239-supplementary.pdf]

Electronic Supporting Information for:

A quantum chemical deep-dive into the π-π interactions of 3-methylindole and its halogenated derivatives – towards an improved ligand design and tryptophan stacking

Ruben Van Lommel [1,2,*], Tom Bettens [1], Thomas M. A. Barlow [3], Jolien Bertouille [3], Steven Ballet [3,*] and Frank De Proft [1,*]

[1] Eenheid Algemene Chemie (ALGC), Faculty of Science and Bio-engineering Sciences, Vrije Universiteit Brussel (VUB), Pleinlaan 2, 1050 Brussels, Belgium.
[2] Molecular Design and Synthesis, Department of Chemistry, KU Leuven, Celestijnenlaan 200F Leuven Chem&Tech, box 2404, 3001 Leuven, Belgium.
[3] Research Group of Organic Chemistry (ORGC), Faculty of Science and Bio-engineering Sciences, Vrije Universiteit Brussel (VUB), Pleinlaan 2, 1050 Brussels, Belgium.
* Correspondence: ruben.vanlommel@kuleuven.be, steven.ballet@vub.be, fdeprof@vub.be

Electronic Supporting Information

- Table of contents -

1. **Energy decomposition analyses** 3

2. **Monomer descriptors** 4

3. **Model optimization and performance statistics** 5

4. **Cartesian coordinates** 9

1. Energy decomposition analyses

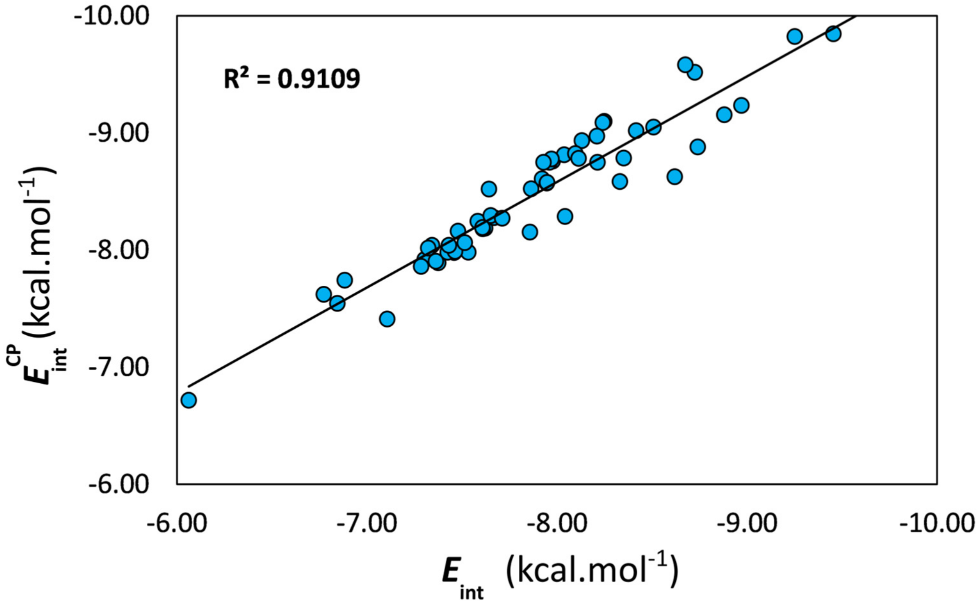

Figure S1. Correlation between counterpoise-corrected interaction energies (E_{int}^{CP}) and the interaction energy stemming from the energies decomposition analyses (E_{int}).

Table S1. Results of the energy decomposition analyses of the 3-methylindole dimers set. In this table, the interaction energy ($\boldsymbol{\Delta}E_{int}$), the electrostatic ($\boldsymbol{\Delta}V_{elstat}$), Pauli repulsion ($\boldsymbol{\Delta}E_{Pauli}$) and orbital interaction ($\boldsymbol{\Delta}E_{oi}$) terms have been computed at the M06/TZ2P(ZORA) level of theory and expressed relative to the parent ***H-H*** dimer for comparative purposes. All calculations were performed in the gas phase and values are expressed in kcal.mol^{-1}.

Dimer	ΔE_{int} (kcal.mol^{-1})	ΔV_{elstat} (kcal.mol^{-1})	ΔE_{Pauli} (kcal.mol^{-1})	ΔE_{oi} (kcal.mol^{-1})
H-H	0.00	0.00	0.00	0.00
H-4F	-0.06	-0.42	0.65	-0.29
H-4Cl	-0.57	-0.65	0.50	-0.41
H-4Br	-0.63	-0.70	0.60	-0.52
H-4I	-0.83	-0.92	0.67	-0.58
H-5F	0.21	0.00	0.25	-0.05
H-5Cl	0.00	-0.07	0.12	-0.04
H-5Br	-0.09	-0.29	0.41	-0.20
H-5I	0.03	-0.38	0.47	-0.06
H-6F	0.29	-0.26	0.80	-0.25
H-6Cl	-0.11	-0.76	1.01	-0.36
H-6Br	-0.42	-1.02	1.14	-0.53
H-6I	-0.60	-0.93	0.74	-0.41
H-7F	0.15	0.02	0.26	-0.12
H-7Cl	0.27	-0.09	0.48	-0.12
H-7Br	0.21	0.27	0.03	-0.09
H-7I	0.20	0.30	-0.12	0.02
4F-4F	0.03	-0.02	0.12	-0.07
4Cl-4Cl	-0.56	-1.08	0.96	-0.43
4Br-4Br	-0.91	-0.93	0.66	-0.64
4I-4I	-1.64	-1.94	1.43	-1.13
4F-5F	0.20	-0.15	0.55	-0.19
4Cl-5Cl	-0.56	-1.08	0.96	-0.43
4Br-5Br	-0.59	-1.12	1.19	-0.66
4I-5I	-0.86	-1.60	1.54	-0.80
4F-6F	0.28	-0.17	0.63	-0.18
4Cl-6Cl	-0.56	-1.16	1.17	-0.56
4Br-6Br	-0.75	-1.20	1.18	-0.72
4I-6I	-1.05	-1.68	1.28	-0.64

4F-7F	0.17	-1.13	2.03	-0.74
4Cl-7Cl	-0.34	-0.54	0.47	-0.26
4Br-7Br	-0.64	-0.65	0.47	-0.45
4I-7I	-0.69	-0.68	0.27	-0.29
5F-5F	1.47	1.16	0.16	0.15
5Cl-5Cl	-0.60	0.02	-0.43	-0.18
5Br-5Br	-1.33	-0.19	-0.57	-0.57
5I-5I	-1.83	-0.72	-0.43	-0.68
5F-6F	0.78	0.47	0.25	0.06
5Cl-6Cl	0.15	-0.41	0.73	-0.17
5Br-6Br	-0.33	-0.80	0.96	-0.49
5I-6I	-0.40	-0.76	0.64	-0.27
5F-7F	0.33	0.00	0.50	-0.17
5Cl-7Cl	0.13	0.10	0.09	-0.06
5Br-7Br	0.00	0.14	0.03	-0.17
5I-7I	-0.10	0.20	-0.19	-0.11
6F-6F	0.64	-0.43	1.40	-0.32
6Cl-6Cl	-0.39	-0.71	0.69	-0.36
6Br-6Br	-0.90	-1.40	1.38	-0.89
6I-6I	-0.97	-1.12	0.70	-0.55
6F-7F	0.44	-0.53	1.35	-0.38
6Cl-7Cl	-0.79	-1.43	1.44	-0.79
6Br-7Br	-1.40	-1.69	1.44	-1.15
6I-7I	-1.66	-1.79	1.26	-1.13
7F-7F	0.57	-0.19	1.12	-0.36
7Cl-7Cl	-0.09	-0.18	0.48	-0.38
7Br-7Br	-0.39	0.09	0.10	-0.57
7I-7I	-0.44	-0.29	0.21	-0.36

2. Monomer descriptors

Table S2. Values of the computed descriptors of the 3-methylindole monomers (#).

#	IE (a.u.)	EA (a.u.)	μ (D)	α (a.u.)	η (a.u.)	S (a.u.)	ESP_{max} (kcal/mol)	ESP_{min} (kcal/mol)	s^0_{max} (a.u.)	s^+_{max} (a.u.)	s^-_{max} (a.u.)	$α_{zz}$ (a.u.)
**3-methylindole derivatives**												
H	0.2735	-0.0512	2.01	107.06	0.1631	6.13	2.35	-11.19	-2.35E-05	6.08E-02	3.73E-02	56.77
4F	0.2758	-0.0516	3.04	106.73	0.1643	6.09	3.66	-8.87	1.46E-05	3.23E-02	3.02E-02	56.56
4Cl	0.2756	-0.0390	3.46	120.07	0.1581	6.33	3.03	-9.21	5.85E-02	2.86E-02	3.00E-02	62.10
4Br	0.2744	-0.0363	3.50	127.20	0.1561	6.41	4.28	-9.85	8.00E-02	4.18E-02	4.28E-02	65.55
4I	0.2698	-0.0336	3.43	140.45	0.1521	6.57	4.03	-10.37	1.85E-01	7.40E-02	1.12E-01	75.52
5F	0.2799	-0.0424	3.53	107.16	0.1616	6.19	4.69	-8.70	8.79E-02	5.12E-02	3.67E-02	56.75
5Cl	0.2806	-0.0366	4.03	121.53	0.1590	6.29	5.77	-9.89	8.57E-02	5.09E-02	3.49E-02	62.42
5Br	0.2796	-0.0349	4.06	129.06	0.1575	6.35	5.86	-10.59	8.40E-02	5.04E-02	3.37E-02	66.06
5I	0.2753	-0.0333	3.93	142.98	0.1539	6.50	5.81	-11.08	1.66E-01	5.18E-02	1.14E-01	76.50
6F	0.2773	-0.0460	2.83	107.03	0.1623	6.16	3.25	-8.51	1.07E-01	7.02E-02	3.67E-02	56.82
6Cl	0.2774	-0.0371	3.31	122.40	0.1578	6.34	5.13	-8.98	8.95E-02	5.40E-02	3.57E-02	62.53
6Br	0.2761	-0.0347	3.34	130.17	0.1558	6.42	3.98	-9.67	8.58E-02	5.09E-02	3.85E-02	66.20
6I	0.2715	-0.0325	3.23	144.43	0.1520	6.58	4.05	-10.13	1.83E-01	6.53E-02	1.18E-01	76.61
7F	0.2782	-0.0506	1.73	107.00	0.1650	6.06	2.28	-8.89	9.46E-02	5.99E-02	3.47E-02	56.64
7Cl	0.2790	-0.0379	1.78	120.44	0.1591	6.28	1.85	-8.45	8.75E-02	5.26E-02	3.49E-02	62.15
7Br	0.2781	-0.0353	1.76	127.52	0.1574	6.35	1.66	-8.47	8.54E-02	5.08E-02	3.47E-02	65.83
7I	0.2747	-0.0328	1.64	141.18	0.1543	6.48	1.74	-8.70	1.65E-01	7.37E-02	9.17E-02	76.17

Table S3. Values of the computed descriptors of the toluene monomers (#)

#	IE (a.u.)	α (a.u.)	s^0_{max} (a.u.)
**Toluene derivatives**			
H	0.3205	76.39	2.92E-02
*o*F	0.3235	76.37	6.20E-02
*m*F	0.3252	76.56	6.84E-02
*p*F	0.3190	76.53	3.07E-02
*o*Cl	0.3209	89.22	6.06E-02
*m*Cl	0.3211	90.06	2.69E-02
*p*Cl	0.3156	90.48	2.95E-02
*o*Br	0.3167	96.09	6.96E-02
*m*Br	0.3161	97.14	8.74E-02
*p*Br	0.3111	97.68	3.81E-02
*o*I	0.3055	109.34	1.42E-01
*m*I	0.3045	110.76	1.01E-01
*p*I	0.3002	111.58	1.22E-01

3. Model optimization and performance statistics

Table S4. Dimer descriptors are calculated as the sum of the descriptor values of the respective monomers. This table collects the dimer descriptors calculated for the 3-methylindole dimers used to construct the model. Dimers that were excluded during the training of the model, but used during validation are annotated with the letter [v].

#	∑IE (a.u.)	∑EA (a.u.)	∑μ (D)	∑α (a.u.)	∑η (a.u.)	∑S (a.u.)	$\sum ESP_{max}$ (kcal/mol)	$\sum ESP_{min}$ (kcal/mol)	$\sum s^0_{max}$ (a.u.)	$\sum s^+_{max}$ (a.u.)	$\sum s^-_{max}$ (a.u.)	$\sum \alpha_{zz}$ (a.u.)
**3-methylindole dimers**												
H-H[v]	0.5471	-0.1023	4.02	214.12	0.3263	12.26	4.70	-22.39	-4.69E-05	1.22E-01	7.46E-02	113.54
H-4F	0.5494	-0.1027	5.05	213.79	0.3275	12.22	6.01	-20.07	-8.83E-06	9.30E-02	6.75E-02	113.33
H-4Cl	0.5491	-0.0902	5.47	227.13	0.3212	12.46	5.38	-20.40	5.85E-02	8.94E-02	6.73E-02	118.87
H-Br	0.5479	-0.0874	5.51	234.25	0.3192	12.54	6.63	-21.05	7.99E-02	1.03E-01	8.02E-02	122.32
H-I	0.5434	-0.0847	5.44	247.50	0.3153	12.70	6.38	-21.56	1.85E-01	1.35E-01	1.49E-01	132.29
H-5F	0.5535	-0.0935	5.54	214.22	0.3248	12.32	7.04	-19.89	8.78E-02	1.12E-01	7.40E-02	113.52
H-5Cl	0.5541	-0.0878	6.04	228.59	0.3222	12.42	8.12	-21.08	8.57E-02	1.12E-01	7.22E-02	119.18
H-5Br	0.5532	-0.0861	6.07	236.12	0.3207	12.48	8.21	-21.79	8.40E-02	1.11E-01	7.11E-02	122.83
H-5I[v]	0.5488	-0.0845	5.94	250.04	0.3171	12.63	8.16	-22.28	1.66E-01	1.13E-01	1.52E-01	133.27
H-6F	0.5508	-0.0972	4.84	214.09	0.3255	12.29	5.60	-19.70	1.07E-01	1.31E-01	7.40E-02	113.58
H-6Cl	0.5509	-0.0883	5.32	229.46	0.3209	12.47	7.48	-20.17	8.95E-02	1.15E-01	7.30E-02	119.29
H-6Br	0.5497	-0.0859	5.35	237.23	0.3190	12.55	6.33	-20.87	8.58E-02	1.12E-01	7.58E-02	122.97
H-6I	0.5450	-0.0836	5.23	251.49	0.3151	12.71	6.40	-21.32	1.83E-01	1.26E-01	1.56E-01	133.38
H-7F	0.5518	-0.1018	3.74	214.06	0.3281	12.19	4.63	-20.09	9.45E-02	1.21E-01	7.20E-02	113.41
H-7Cl	0.5525	-0.0891	3.79	227.50	0.3223	12.41	4.20	-19.65	8.75E-02	1.13E-01	7.22E-02	118.92
H-7Br	0.5516	-0.0864	3.76	234.57	0.3205	12.48	4.01	-19.67	8.54E-02	1.12E-01	7.20E-02	122.60
H-7I[v]	0.5483	-0.0840	3.64	248.24	0.3174	12.61	4.09	-19.90	1.65E-01	1.34E-01	1.29E-01	132.94
4F-4F	0.5517	-0.1031	6.09	213.46	0.3287	12.17	7.32	-17.75	2.93E-05	6.45E-02	6.03E-02	113.12
4Cl-4Cl[v]	0.5512	-0.0780	6.93	240.14	0.3162	12.65	6.06	-18.41	1.17E-01	5.73E-02	5.99E-02	124.19
4Br-4Br	0.5487	-0.0725	7.00	254.39	0.3121	12.82	8.57	-19.71	1.60E-01	8.37E-02	8.57E-02	131.10

4I-4I	0.5397	-0.0671	6.86	280.89	0.3042	13.15	8.07	-20.74	3.71E-01	1.48E-01	2.23E-01	151.04
4F-5F	0.5558	-0.0939	6.57	213.89	0.3260	12.27	8.35	-17.57	8.79E-02	8.34E-02	6.69E-02	113.31
4Cl-5Cl	0.5562	-0.0757	7.50	241.60	0.3171	12.61	8.80	-19.09	1.44E-01	7.96E-02	6.49E-02	124.51
4Br-5Br[v]	0.5540	-0.0712	7.56	256.26	0.3136	12.76	10.14	-20.45	1.64E-01	9.23E-02	7.66E-02	131.61
4I-5I	0.5451	-0.0669	7.36	283.43	0.3060	13.07	9.84	-21.45	3.51E-01	1.26E-01	2.26E-01	152.02
4F-6F[v]	0.5531	-0.0976	5.87	213.77	0.3267	12.25	6.91	-17.38	1.07E-01	1.02E-01	6.69E-02	113.38
4Cl-6Cl[v]	0.5530	-0.0761	6.77	242.47	0.3159	12.66	8.16	-18.18	1.48E-01	8.26E-02	6.56E-02	124.62
4Br-6Br[v]	0.5505	-0.0710	6.84	257.37	0.3119	12.82	8.26	-19.53	1.66E-01	9.28E-02	8.13E-02	131.75
4I-6I[v]	0.5413	-0.0660	6.66	284.88	0.3041	13.15	8.08	-20.50	3.69E-01	1.39E-01	2.30E-01	152.13
4F-7F[v]	0.5541	-0.1022	4.77	213.73	0.3293	12.15	5.94	-17.77	9.46E-02	9.22E-02	6.49E-02	113.20
4Cl-7Cl	0.5546	-0.0769	5.24	240.51	0.3172	12.61	4.88	-17.66	1.46E-01	8.13E-02	6.49E-02	124.25
4Br-7Br	0.5525	-0.0715	5.26	254.71	0.3134	12.76	5.95	-18.32	1.65E-01	9.27E-02	7.75E-02	131.38
4I-7I[v]	0.5446	-0.0664	5.07	281.62	0.3064	13.05	5.78	-19.07	3.50E-01	1.48E-01	2.03E-01	151.70
5F-5F	0.5598	-0.0847	7.06	214.33	0.3233	12.37	9.37	-17.39	1.76E-01	1.02E-01	7.35E-02	113.50
5Cl-5Cl	0.5612	-0.0733	8.07	243.06	0.3181	12.58	11.55	-19.77	1.71E-01	1.02E-01	6.99E-02	124.83
5Br-5Br	0.5593	-0.0698	8.12	258.13	0.3151	12.70	11.72	-21.19	1.68E-01	1.01E-01	6.75E-02	132.13
5I-5I	0.5506	-0.0666	7.86	285.96	0.3078	12.99	11.62	-22.17	3.32E-01	1.04E-01	2.29E-01	153.00
5F-6F	0.5572	-0.0884	6.36	214.20	0.3240	12.35	7.94	-17.21	1.95E-01	1.21E-01	7.35E-02	113.57
5Cl-6Cl[v]	0.5580	-0.0738	7.35	243.93	0.3168	12.63	10.91	-18.86	1.75E-01	1.05E-01	7.06E-02	124.94
5Br-6Br	0.5558	-0.0696	7.40	259.23	0.3134	12.76	9.84	-20.27	1.70E-01	1.01E-01	7.23E-02	132.26
5I-6I	0.5468	-0.0658	7.15	287.41	0.3059	13.08	9.85	-21.21	3.49E-01	1.17E-01	2.33E-01	153.11
5F-7F	0.5582	-0.0930	5.26	214.16	0.3266	12.25	6.97	-17.59	1.82E-01	1.11E-01	7.14E-02	113.39
5Cl-7Cl	0.5596	-0.0746	5.81	241.97	0.3182	12.57	7.63	-18.34	1.73E-01	1.04E-01	6.99E-02	124.57
5Br-7Br	0.5577	-0.0702	5.82	256.58	0.3149	12.70	7.52	-19.07	1.69E-01	1.01E-01	6.84E-02	131.89
5I-7I	0.5500	-0.0661	5.57	284.16	0.3082	12.98	7.55	-19.79	3.31E-01	1.25E-01	2.06E-01	152.67
6F-6F	0.5545	-0.0921	5.66	214.07	0.3247	12.32	6.51	-17.02	2.14E-01	1.40E-01	7.35E-02	113.63
6Cl-6Cl	0.5547	-0.0742	6.62	244.81	0.3156	12.68	10.27	-17.95	1.79E-01	1.08E-01	7.13E-02	125.05
6Br-6Br	0.5523	-0.0695	6.68	260.34	0.3117	12.83	7.95	-19.35	1.72E-01	1.02E-01	7.70E-02	132.40
6I-6I	0.5429	-0.0649	6.45	288.86	0.3040	13.16	8.09	-20.26	3.67E-01	1.31E-01	2.37E-01	153.22
6F-7F	0.5555	-0.0966	4.56	214.03	0.3273	12.22	5.54	-17.40	2.01E-01	1.30E-01	7.15E-02	113.45
6Cl-7Cl	0.5563	-0.0750	5.09	242.84	0.3169	12.62	6.99	-17.43	1.77E-01	1.07E-01	7.06E-02	124.68
6Br-7Br	0.5542	-0.0700	5.10	257.68	0.3132	12.77	5.64	-18.15	1.71E-01	1.02E-01	7.32E-02	132.03
6I-7I	0.5462	-0.0653	4.86	285.61	0.3063	13.06	5.79	-18.83	3.48E-01	1.39E-01	2.10E-01	152.78
7F-7F[v]	0.5565	-0.1012	3.46	213.99	0.3300	12.12	4.56	-17.79	1.89E-01	1.20E-01	6.94E-02	113.28
7Cl-7Cl	0.5579	-0.0758	3.56	240.88	0.3183	12.57	3.71	-16.90	1.75E-01	1.05E-01	6.99E-02	124.30
7Br-7Br	0.5562	-0.0705	3.51	255.03	0.3148	12.71	3.32	-16.94	1.71E-01	1.02E-01	6.94E-02	131.66
7I-7I[v]	0.5495	-0.0656	3.27	282.36	0.3086	12.96	3.49	-17.41	3.30E-01	1.47E-01	1.83E-01	152.35

Table S5. Parameter coefficient estimates, standard error and p-value for the descriptors present in the optimized model.

Descriptor/term	Estimate	Standard error	t ratio	p-value
Intercept	-18.09876	7.602228	-2.38	0.0223
IE	29.501211	13.12205	2.25	0.0303
α	-0.026361	0.004152	-6.35	<.0001
s^0_{max}	1.7908569	1.008514	1.78	0.0836

Table S6. The explicitly calculated interaction energies of the 3-methylindole dimers and the predicted energies originating from the optimized model, both provided in kcal.mol^{-1}.

Dimer	E_{int}^{CP} (kcal.mol^{-1})	E_{int}^{Pred} (kcal.mol^{-1})
H-H	-7.62	-7.60
H-4F	-7.58	-7.53
H-4Cl	-7.98	-7.78
H-4Br	-8.04	-7.97
H-4I	-8.42	-8.26
H-5F	-7.46	-7.26
H-5Cl	-7.61	-7.62
H-5Br	-7.67	-7.85
H-5I	-7.86	-8.20
H-6F	-7.38	-7.30
H-6Cl	-7.65	-7.74
H-6Br	-7.92	-7.98
H-6I	-8.35	-8.32
H-7F	-7.34	-7.29
H-7Cl	-7.30	-7.64
H-7Br	-7.42	-7.86
H-7I	-7.53	-8.17
4F-4F	-7.48	-7.45
4Cl-4Cl	-8.21	-7.96
4Br-4Br	-8.25	-8.33
4I-4I	-9.25	-8.92
4F-5F	-7.46	-7.18
4Cl-5Cl	-7.96	-7.80
4Br-5Br	-7.97	-8.22
4I-5I	-8.51	-8.86
4F-6F	-7.36	-7.23
4Cl-6Cl	-7.93	-7.91
4Br-6Br	-8.13	-8.35
4I-6I	-8.97	-8.98
4F-7F	-7.32	-7.22
4Cl-7Cl	-7.86	-7.82
4Br-7Br	-8.10	-8.22
4I-7I	-8.74	-8.83
5F-5F	-6.06	-6.92
5Cl-5Cl	-8.11	-7.64
5Br-5Br	-8.73	-8.10
5I-5I	-9.74	-8.80
5F-6F	-7.11	-6.96
5Cl-6Cl	-7.43	-7.75
5Br-6Br	-7.64	-8.23
5I-6I	-8.33	-8.92
5F-7F	-7.28	-6.95
5Cl-7Cl	-7.51	-7.66
5Br-7Br	-7.61	-8.11
5I-7I	-8.04	-8.77
6F-6F	-6.84	-7.00
6Cl-6Cl	-7.95	-7.87
6Br-6Br	-8.24	-8.36
6I-6I	-8.88	-9.04
6F-7F	-6.88	-6.99
6Cl-7Cl	-8.21	-7.77
6Br-7Br	-8.68	-8.23
6I-7I	-9.45	-8.89
7F-7F	-6.77	-6.98
7Cl-7Cl	-7.71	-7.68
7Br-7Br	-7.95	-8.11
7I-7I	-8.62	-8.74

Table S7. Dimer descriptors are calculated as the sum of the descriptor values of the respective monomers. This table collects the dimer descriptors calculated for the cross-halogenated 3-methylindole dimers and toluene dimers used to test the applicability window of the model. The explicitly calculated interaction energies and predicted energies are also provided in kcal.mol^{-1}.

#	IE (a.u.)	α (a.u.)	s^0_{max} (a.u.)	E_{int}^{CP} (kcal.mol^{-1})	E_{int}^{Pred} (kcal.mol^{-1})
**Cross-halogenated 3-methylindole dimers**					
4I-7Cl	0.5488	260.89	2.73E-01	-8.42	-8.30
5Cl-6F	0.5579	228.56	1.92E-01	-7.25	-7.33
5Cl-7Br	0.5587	249.04	1.71E-01	-7.63	-7.88
6F-6Br	0.5534	237.20	1.93E-01	-8.12	-7.68
4F-6I	0.5473	251.16	1.83E-01	-8.81	-8.25
**Toluene dimers**					
H-H	0.6410	152.78	5.85E-02	-3.63	-3.11
H-*o*F	0.6440	152.75	9.13E-02	-3.6	-2.96
H-*m*F	0.6457	152.95	9.77E-02	-3.48	-2.91
H-*p*F	0.6395	152.92	5.99E-02	-3.6	-3.16
H-*o*Cl	0.6414	165.61	8.98E-02	-3.94	-3.38
H-*m*Cl	0.6416	166.45	5.62E-02	-3.95	-3.46
H-*p*Cl	0.6361	166.87	5.87E-02	-4.11	-3.63
H-*o*Br	0.6372	172.48	9.89E-02	-4.08	-3.67
H-*m*Br	0.6366	173.53	1.17E-01	-4.21	-3.68
H-*p*Br	0.6316	174.07	6.73E-02	-4.27	-3.93
H-*o*I	0.6261	185.73	1.71E-01	-4.15	-4.22
H-*m*I	0.6250	187.15	1.30E-01	-4.33	-4.36
H-*p*I	0.6207	187.96	1.52E-01	-4.52	-4.47
*m*F-*m*F	0.6503	153.13	1.37E-01	-4.36	-2.71
*m*F-*p*F	0.6441	153.10	9.91E-02	-4.12	-2.95
*m*F-*o*F	0.6486	152.93	1.30E-01	-3.99	-2.76
*o*F-*o*F	0.6503	153.13	1.37E-01	-3.65	-2.71
*o*F-*p*F	0.6461	165.79	1.29E-01	-3.86	-3.18
*p*F-*p*F	0.6418	178.45	1.21E-01	-3.65	-3.65
*m*Cl-*m*Cl	0.6422	180.12	5.38E-02	-6.52	-3.81
*m*Cl-*p*Cl	0.6367	180.54	5.64E-02	-5.21	-3.97
*m*Cl-*o*Cl	0.6420	179.28	8.75E-02	-4.82	-3.73
*o*Cl-*o*Cl	0.6418	178.45	1.21E-01	-4.51	-3.65
*o*Cl-*p*Cl	0.6365	179.70	9.01E-02	-4.92	-3.90
*p*Cl-*p*Cl	0.6311	180.95	5.90E-02	-4.56	-4.14
*m*Br-*m*Br	0.6322	194.28	1.75E-01	-6.21	-4.26
*m*Br-*p*Br	0.6272	194.82	1.25E-01	-5.59	-4.51
*m*Br-*o*Br	0.6328	193.23	1.57E-01	-5.18	-4.24
*o*Br-*o*Br	0.6334	192.18	1.39E-01	-4.57	-4.23
*o*Br-*p*Br	0.6278	193.77	1.08E-01	-5.34	-4.49
*p*Br-*p*Br	0.6222	195.37	7.61E-02	-4.75	-4.76
*m*I-*m*I	0.6089	221.51	2.02E-01	-6.55	-5.61
*m*I-*p*I	0.6046	222.33	2.23E-01	-5.85	-5.72
*m*I-*o*I	0.6100	220.10	2.43E-01	-6.15	-5.47

*o*I-*o*I	0.6111	218.69	2.84E-01	-5.36	-5.33
*o*I-*p*I	0.6057	220.92	2.64E-01	-5.72	-5.58
*o*I-*p*I	0.6003	223.15	2.45E-01	-5.42	-5.83

4. Cartesian coordinates

Coordinates of 3-methylindole monomers

Electronic energies are provided at M06/cc-PVTZ(-PP) (and M06/aug-cc-PVTZ(-PP)) level of theory.

All structures are characterized by 0 imaginary frequencies.

Entry: H

E_{elec} (a.u.): -402.95454 (-402.96157)

E_{elec} N-1 system (a.u.): -402.68100

E_{elec} N+1 system (a.u.): -402.90337

C	-1.59395100	-0.22266500	0.00000000
C	-1.82597900	1.11682500	0.00000100
C	-0.17209000	-0.38351800	0.00000000
H	-2.76627800	1.64484300	0.00000200
N	-0.63240900	1.80545100	-0.00000100
C	0.39965000	0.90439000	0.00000000
C	0.66462200	-1.49882400	0.00000000
H	-0.53604800	2.80258400	-0.00000100
C	1.77392700	1.10005000	0.00000000
C	2.02886400	-1.30883300	0.00000000
H	0.24345100	-2.49761600	0.00000000
H	2.20195300	2.09490100	-0.00000100
C	2.57654600	-0.02092100	0.00000000
H	2.69181200	-2.16433900	0.00000000
H	3.65254000	0.09641500	0.00000000
C	-2.59650000	-1.31570300	-0.00000100
H	-2.48896600	-1.95874700	-0.87730900
H	-2.48896500	-1.95874900	0.87730600
H	-3.61316900	-0.92225400	0.00000100

Entry: 4F

E_{elec} (a.u.): -502.19948 (-502.20804)

E_{elec} N-1 system (a.u.): -501.92365

E_{elec} N+1 system (a.u.): -502.14791

C	1.61922100	0.03302900	0.00000000
C	1.90919400	-1.29514600	0.00000000
C	0.18965300	0.11912900	0.00000100
H	2.87442700	-1.77591300	-0.00000100
N	0.75014500	-2.03920600	0.00000000
C	-0.32486800	-1.19306800	0.00000000
C	-0.73138000	1.16167100	0.00000200
H	0.69942800	-3.03984500	0.00000000
C	-1.68830100	-1.46727400	0.00000000
C	-2.08076700	0.92325900	0.00000000
H	-2.05716200	-2.48470700	0.00000000
C	-2.55126900	-0.39479000	-0.00000100
H	-2.76270500	1.76241200	-0.00000100
H	-3.61923700	-0.56798000	-0.00000200
C	2.59404900	1.15286700	0.00000000
H	2.47386700	1.79341000	-0.87576500
H	2.47387000	1.79340700	0.87576800
H	3.61643500	0.77395800	-0.00000300
F	-0.28479200	2.42018100	-0.00000100

Entry: 4Cl

E_{elec} (a.u.): -862.55901 (-862.56633)

E_{elec} N-1 system (a.u.): -862.28340

E_{elec} N+1 system (a.u.): -862.51998

C	-0.76023300	-1.46650600	0.00000200
C	-2.11296500	-1.32567200	-0.00000300
C	-0.22617800	-0.13559900	0.00000300
H	-2.86902100	-2.09435600	-0.00000500
N	-2.45942200	0.00467900	-0.00000200
C	-1.32022300	0.76040900	0.00000100
C	1.05043800	0.42764900	0.00000300
H	-3.39431400	0.36569800	-0.00000400
C	-1.16894700	2.14048300	0.00000100
C	1.22238200	1.79133000	0.00000100
H	-2.02925300	2.79748100	-0.00000100
C	0.11290100	2.64094800	0.00000000
H	2.22441700	2.19757800	-0.00000100
H	0.27179800	3.71110900	-0.00000200
C	-0.03352200	-2.76321900	0.00000200
H	0.60754400	-2.87304800	-0.87608800
H	0.60749200	-2.87307800	0.87612500
H	-0.74279800	-3.59190700	-0.00003200
Cl	2.46812800	-0.59359800	-0.00000200

Entry: 4Br

E_{elec} (a.u.): -2976.46495 (-2976.47208)

E_{elec} N-1 system (a.u.): -2976.19058

E_{elec} N+1 system (a.u.): -2976.42869

C	0.89340800	1.59000200	0.00000000
C	2.24302400	1.75974900	0.00000100
C	0.67338700	0.17109800	-0.00000100
H	2.80370900	2.68071400	0.00000200
N	2.88286000	0.54425200	0.00000000
C	1.94531400	-0.44995600	0.00000000
C	-0.43831400	-0.67420100	-0.00000100
H	3.87520700	0.40513700	0.00000100
C	2.11884900	-1.82674200	0.00000000
C	-0.28678200	-2.04119000	0.00000000
H	3.10882600	-2.26492400	0.00000000
C	0.98911800	-2.61153300	0.00000000
H	-1.16352200	-2.67427500	0.00000000
H	1.08197800	-3.68940600	0.00000000
C	-0.09806700	2.69798900	-0.00000100
H	-0.74799000	2.66767700	-0.87598200
H	-0.74800600	2.66766300	0.87596800
H	0.41715300	3.65957800	0.00001100
Br	-2.20134300	0.02990800	0.00000000

Entry: 4I

E_{elec} (a.u.): -698.12204 (-698.12898)

E_{elec} N-1 system (a.u.): -697.85222

E_{elec} N+1 system (a.u.): -698.08848

C	1.29138100	1.59519100	0.00000100
C	2.63858800	1.78303500	-0.00000200

Entry: 5F

E_{elec} (a.u.): -502.19868 (-502.20763)

E_{elec} N-1 system (a.u.): -501.91875

E_{elec} N+1 system (a.u.): -502.15633

C	-1.79624400	-0.57168900	0.00000000
C	-2.43429000	0.63043400	0.00000000

C	1.08616000	0.17254900	0.00000200
H	3.18663000	2.71160700	-0.00000300
N	3.29399100	0.57709400	-0.00000300
C	2.36951200	-0.42889800	-0.00000100
C	-0.01532300	-0.69123300	0.00000500
H	4.28797700	0.44988800	-0.00000300
C	2.56904300	-1.80153200	-0.00000100
C	0.16977200	-2.05670400	0.00000300
H	3.56735700	-2.22053300	-0.00000300
C	1.45422200	-2.60642800	0.00000100
H	-0.68993000	-2.71280100	0.00000400
H	1.56403700	-3.68281800	0.00000000
C	0.29276800	2.69635300	0.00000400
H	-0.35737200	2.66255400	-0.87576900
H	-0.35735200	2.66256700	0.87579200
H	0.80292200	3.66081800	-0.00000900
I	-2.00375400	0.00858600	-0.00000100

Entry: 5Cl
E_{elec} (a.u.): -862.55903 (-862.56645)
E_{elec} N-1 system (a.u.): -862.27842
E_{elec} N+1 system (a.u.): -862.52238

C	2.04356500	-0.78783300	0.00000000
C	2.88545200	0.28026000	-0.00000100
C	0.71898300	-0.24977600	0.00000000
H	3.96351000	0.29574600	-0.00000100
N	2.16582200	1.45436200	0.00000000
C	0.82929900	1.15482800	0.00000000
C	-0.54358900	-0.84072900	0.00000000
H	2.55762400	2.37664300	0.00000000
C	-0.28801400	1.97759800	0.00000000
C	-1.63999700	-0.01304000	0.00000000
H	-0.19716400	3.05631300	0.00000000
C	-1.52925800	1.37941700	0.00000000
H	-2.42922900	1.97833500	0.00000000
C	2.39937800	-2.22761600	0.00000000
H	1.99562400	-2.73914900	0.87741500
H	1.99562400	-2.73914900	-0.87741500
H	3.47981700	-2.37040800	0.00000100
Cl	-3.24216500	-0.71536200	0.00000000
H	-0.66466400	-1.91636200	0.00000000

Entry: 5I
E_{elec} (a.u.): -698.12326 (-698.13015)
E_{elec} N-1 system (a.u.): -697.84797
E_{elec} N+1 system (a.u.): -698.08993

C	3.04092700	-0.95646800	0.00000000
C	4.02559600	-0.01897600	-0.00000400
C	1.80554000	-0.23568400	0.00000000
H	5.09510200	-0.15604500	-0.00000600
N	3.47967100	1.24588300	-0.00000100
C	2.11511800	1.13928600	-0.00000100
C	0.47085800	-0.63878800	0.00000100
H	3.99829700	2.10327100	-0.00000300
C	1.12699000	2.11330300	0.00000000
C	-0.50262200	0.33448700	0.00000800
H	1.37083200	3.16822200	0.00000100
C	-0.18723700	1.69784100	0.00000200
H	-0.98302700	2.43010300	0.00000800
C	3.19042800	-2.43195700	0.00000200
H	2.71930600	-2.88220800	0.87743600
H	2.71930500	-2.88221000	-0.87743100
H	4.24010800	-2.72526000	0.00000200
I	-2.53286800	-0.22839800	-0.00000100
H	0.21079400	-1.69024600	0.00000600

Entry: 6Cl
E_{elec} (a.u.): -862.55914 (-862.56656)
E_{elec} N-1 system (a.u.): -862.28178
E_{elec} N+1 system (a.u.): -862.52203

C	-0.39682900	-0.28087300	0.00000000
H	-3.49302600	0.83573300	0.00000000
N	-1.51911300	1.65620000	0.00000000
C	-0.25552800	1.12135000	0.00000000
C	0.74365400	-1.08582200	0.00000100
H	-1.73883500	2.63361800	0.00000000
C	0.98829300	1.73567700	0.00000000
C	1.96147700	-0.45756900	0.00000000
H	1.08616400	2.81380600	0.00000000
C	2.10523300	0.92844500	0.00000000
H	3.10280300	1.34578900	0.00000000
C	-2.41133600	-1.92120600	0.00000000
H	-2.11165400	-2.49989800	-0.87753700
H	-2.11165100	-2.49989900	0.87753500
H	-3.49962100	-1.86005100	0.00000100
F	3.07592400	-1.19866800	0.00000000
H	0.68971100	-2.16697600	0.00000000

Entry: 5Br
E_{elec} (a.u.): -2976.46550 (-2976.47266)
E_{elec} N-1 system (a.u.): -2976.18586
E_{elec} N+1 system (a.u.): -2976.43060

C	2.57422100	-0.90218800	0.00000000
C	3.51767600	0.07705100	-0.00000100
C	1.30936500	-0.23556000	0.00000000
H	4.59203400	-0.01383800	-0.00000100
N	2.91758600	1.31680500	0.00000000
C	1.55847100	1.15124500	0.00000000
C	-0.00593400	-0.69735600	0.00000000
H	3.39838000	2.19597400	0.00000000
C	0.52860500	2.08140000	0.00000000
C	-1.01619100	0.23485100	0.00000000
H	0.72662100	3.14578500	0.00000000
C	-0.76645700	1.60971500	0.00000000
H	-1.59911000	2.29954400	0.00000000
C	2.78438800	-2.37010800	0.00000000
H	2.33149700	-2.83894600	0.87732200
H	2.33149700	-2.83894600	-0.87732100
H	3.84500100	-2.62098400	0.00000000
Br	-2.82074900	-0.35666900	0.00000000
H	-0.22767200	-1.75712300	0.00000000

Entry: 6F
E_{elec} (a.u.): -502.19913 (-502.20805)
E_{elec} N-1 system (a.u.): -501.92186
E_{elec} N+1 system (a.u.): -502.15308

C	-1.99788400	-0.20428300	0.00000000
C	-2.21043900	1.13688100	0.00000100
C	-0.57742700	-0.38612600	0.00000000
H	-3.14135000	1.68067100	0.00000200
N	-1.00316900	1.80817100	-0.00000100
C	0.01263400	0.89405700	0.00000000
C	0.24200000	-1.51277000	0.00000100
H	-0.89187500	2.80381500	-0.00000100
C	1.39009000	1.07458300	0.00000000
C	1.60983800	-1.35254700	0.00000000
H	1.86330100	2.04708200	0.00000000
C	2.15023700	-0.06871500	0.00000000
C	-3.01458800	-1.28386800	-0.00000100
H	-2.91546800	-1.92800900	-0.87744800
H	-2.91546700	-1.92801000	0.87744500
H	-4.02573000	-0.87688300	0.00000000
H	-0.19257800	-2.50527900	0.00000100
H	2.28414600	-2.19781400	0.00000000
F	3.48115900	0.05155200	-0.00000100

Entry: 6Br
E_{elec} (a.u.): -2976.46552 (-2976.47268)
E_{elec} N-1 system (a.u.): -2976.18939
E_{elec} N+1 system (a.u.): -2976.43078

C	-2.40605500	-0.19030200	0.00000000
C	-2.60204500	1.15452400	0.00000100
C	-0.98920600	-0.38785600	0.00000000
H	-3.52701700	1.70854000	0.00000100
N	-1.38936200	1.81177800	0.00000000
C	-0.38365300	0.88487300	0.00000000
C	-0.18206900	-1.52339100	0.00000000
H	-1.26691300	2.80622100	0.00000000
C	0.99468200	1.04893100	0.00000000
C	1.18694700	-1.37883300	0.00000000
H	1.46633400	2.02225600	0.00000000
C	1.75057900	-0.10084500	0.00000000
C	-3.43570900	-1.25766100	0.00000000
H	-3.34419600	-1.90281700	-0.87744300
H	-3.34419600	-1.90281700	0.87744300
H	-4.44191000	-0.83877000	0.00000000
H	-0.62584000	-2.51188900	0.00000000
H	1.84033500	-2.24002400	0.00000000
Cl	3.49224200	0.04001300	0.00000000

Entry: 6I
E_{elec} (a.u.): -698.12327 (-698.13016)
E_{elec} N-1 system (a.u.): -697.85180
E_{elec} N+1 system (a.u.): -698.09080

C	-3.59687000	-0.17591300	-0.00000100
C	-3.77698400	1.17164800	0.00000000
C	-2.18289500	-0.38947900	0.00000000
H	-4.69597900	1.73565300	0.00000000
N	-2.55794700	1.81486100	0.00000000
C	-1.56228300	0.87594100	0.00000100
C	-1.38786300	-1.53355400	0.00000100
H	-2.42403100	2.80787900	0.00000100
C	-0.18202900	1.02471000	0.00000100
C	-0.01677600	-1.40303300	0.00000200
H	0.28504700	2.00113800	0.00000300
C	0.56832400	-0.13165600	0.00000400
C	-4.63923600	-1.23104200	-0.00000200
H	-4.55537500	-1.87719500	-0.87745000
H	-4.55537400	-1.87719800	0.87744300
H	-5.64043100	-0.80030700	-0.00000100
H	-1.84124900	-2.51790100	0.00000100
H	0.61603700	-2.28027400	0.00000500
I	2.66748400	0.01619700	-0.00000100

Entry: 7Cl
E_{elec} (a.u.): -862.56091 (-862.56846)
E_{elec} N-1 system (a.u.): -862.28194
E_{elec} N+1 system (a.u.): -862.52300

C	2.13020000	-0.42500000	0.00000000
C	1.63826600	-1.69292100	0.00000000
C	0.99605500	0.44789700	0.00000000
H	2.17081300	-2.63039500	0.00000000
N	0.26056900	-1.67014400	0.00000000
C	-0.15077800	-0.37043400	0.00000000
C	0.84741200	1.83427100	0.00000000
H	-0.35061300	-2.46548300	0.00000100
C	-1.42029100	0.18417900	0.00000000
C	-0.42175200	2.36926900	0.00000000
C	-1.55737300	1.55167300	0.00000000
C	3.55288500	-0.00536900	0.00000000
H	3.79211600	0.60074900	-0.87748900
H	3.79211700	0.60074600	0.87749100
H	4.22104100	-0.86654100	-0.00000200
H	1.71817600	2.47881300	0.00000000
H	-0.55515100	3.44298100	0.00000000
Cl	-2.80892800	-0.87152200	0.00000000
H	-2.54844200	1.98463100	0.00000000

C	-3.06242800	-0.18208500	0.00000000
C	-3.24913400	1.16434200	0.00000100
C	-1.64716800	-0.38911400	0.00000000
H	-4.17058900	1.72422400	0.00000200
N	-2.03261700	1.81334700	-0.00000100
C	-1.03313900	0.87943000	0.00000000
C	-0.84745100	-1.52994500	0.00000000
H	-1.90323200	2.80694500	-0.00000100
C	0.34640000	1.03419200	0.00000000
C	0.52299800	-1.39437600	0.00000000
H	0.81873900	2.00731500	-0.00000100
C	1.09610600	-0.12006400	0.00000000
C	-4.10051800	-1.24124900	-0.00000100
H	-4.01444100	-1.88709100	-0.87745900
H	-4.01444000	-1.88709300	0.87745600
H	-5.10320500	-0.81398500	0.00000100
H	-1.29747100	-2.51569300	0.00000000
H	1.16591600	-2.26376500	0.00000100
Br	2.98837300	0.02311200	0.00000000

Entry: 7F
E_{elec} (a.u.): -502.19809 (-502.20714)
E_{elec} N-1 system (a.u.): -501.91984
E_{elec} N+1 system (a.u.): -502.14748

C	1.87994500	-0.10169500	0.00000000
C	1.70463000	-1.45088900	0.00000100
C	0.57147400	0.47764300	0.00000000
H	2.44660400	-2.23315400	0.00000200
N	0.36157400	-1.75739900	0.00000000
C	-0.34505800	-0.58826800	0.00000000
C	0.09939300	1.79221300	0.00000000
H	-0.03850800	-2.67631600	0.00000000
C	-1.70756600	-0.34408500	0.00000000
C	-1.25934500	2.01040700	0.00000000
C	-2.16997700	0.94488300	0.00000000
C	3.15959900	0.64844000	-0.00000100
H	3.24324800	1.29473800	-0.87734500
H	3.24324900	1.29473900	0.87734300
H	4.01693200	-0.02451400	-0.00000100
H	0.79347100	2.62368500	0.00000000
H	-1.64361200	3.02178800	0.00000000
F	-2.55039100	-1.38325100	0.00000000
H	-3.23746200	1.11818100	0.00000000

Entry: 7Br
E_{elec} (a.u.): -2976.46775 (-2976.47503)
E_{elec} N-1 system (a.u.): -2976.18967
E_{elec} N+1 system (a.u.): -2976.43248

C	2.59711600	-0.62122900	0.00000000
C	1.92983200	-1.80607800	-0.00000100
C	1.59835400	0.40358600	0.00000000
H	2.32514900	-2.80913600	-0.00000100
N	0.56972400	-1.58814600	0.00000000
C	0.34527800	-0.24291900	0.00000000
C	1.64797700	1.79668300	0.00000000
H	-0.14820100	-2.28908700	0.00000100
C	-0.83244600	0.48640100	0.00000000
C	0.46850700	2.50825300	0.00000000
C	-0.77175700	1.86031500	0.00000000
C	4.06526200	-0.40969000	0.00000000
H	4.38889500	0.15547000	-0.87764700
H	4.38889500	0.15547100	0.87764700
H	4.60223000	-1.35822900	0.00000200
H	2.60214200	2.31000200	0.00000000
H	0.48962000	3.58991700	0.00000000
Br	-2.49251200	-0.42641900	0.00000000
H	-1.68763000	2.43534000	0.00000000

Entry: 7I
E_{elec} **(a.u.):** -698.12601 (-698.13303)
E_{elec} **N-1 system (a.u.):** -697.85127
E_{elec} **N+1 system (a.u.):** -698.09318

C	3.00608900	-0.71972500	0.00000000
C	2.26257000	-1.85817700	0.00000000
C	2.07622400	0.36735700	0.00000000
H	2.59037000	-2.88521300	0.00000100
N	0.92010800	-1.55051600	-0.00000100
C	0.78069000	-0.19318000	-0.00000100
C	2.22223900	1.75275000	0.00000000
H	0.15920400	-2.20562200	-0.00000100
C	-0.34923200	0.61235700	-0.00000100
C	1.09414600	2.54297400	0.00000000
C	-0.18718200	1.98078900	0.00000000
C	4.48471300	-0.60163800	0.00000000
H	4.84305700	-0.05793300	-0.87772600
H	4.84305600	-0.05793300	0.87772700
H	4.96086100	-1.58208400	0.00000100
H	3.21001400	2.19817600	0.00000100
H	1.18820900	3.62094900	0.00000000
I	-2.25510500	-0.26609400	0.00000000
H	-1.05650200	2.62519500	0.00000000

Coordinates of 3-methylindole dimers

Counterpoise corrected electronic energies are provided at M06/aug-cc-PVTZ(-PP) level of theory.
All structures are characterized by 0 imaginary frequencies

Entry: H-H
E^{CP} **(a.u.):** -805.93529

C	0.42116500	-0.90049200	1.95956800
C	1.35945200	0.83106400	0.91060700
H	-0.28107200	-1.39294700	2.61397500
C	1.99162300	-0.35099600	0.47409500
C	1.70653300	2.07666700	0.40536300
C	2.98893500	-0.27395200	-0.49631500
C	2.68985200	2.12432400	-0.56278400
H	1.21800400	2.97846800	0.75426600
H	3.48414700	-1.17418500	-0.84277500
C	3.32646000	0.95990800	-1.01146700
H	2.98403800	3.08133300	-0.97486900
H	4.09975400	1.03738100	-1.76497000
C	-1.37476100	-1.44433600	-1.15696200
C	-0.42180000	-0.90111800	-1.95958400
C	-1.99164400	-0.35051100	-0.47387000
H	0.28012700	-1.39408700	-2.61393900
N	-0.41993000	0.47151000	-1.83932300
C	-1.35884900	0.83115000	-0.91065400
C	-2.98903700	-0.27274900	0.49639600
H	0.30590700	1.07506700	-2.18311600
C	-1.70544600	2.07703600	-0.40574200
C	-3.32603500	0.96139700	1.01120900
H	-1.21656200	2.97856000	-0.75485400
C	-2.68881500	2.12538200	0.56230600
H	-4.09934700	1.03945200	1.76462900
H	-2.98258200	3.08264600	0.97410500
C	-1.69469300	-2.87792100	-0.95851900
H	-1.48934000	-3.18173300	0.07367200
H	-2.74964300	-3.09017000	-1.14994800
H	-1.10170700	-3.51509700	-1.61540800
H	-3.48472500	-1.17265800	0.84305800
C	1.37393800	-1.44437600	1.15721400
C	1.69320600	-2.87813300	0.95912700
H	1.48725400	-3.18220500	-0.07287000
H	2.74811100	-3.09075300	1.15029400
H	1.10017600	-3.51485000	1.61645600
N	0.42028800	0.47214800	1.83931700
H	-0.30556200	1.07611300	2.18240100

Entry: H-4F
E^{CP} **(a.u.):** -905.18169

C	0.27416100	0.94383300	-1.90657400
C	1.52826700	-0.66825100	-1.00739500
H	-0.53624000	1.35369400	-2.48972400
C	2.01169100	0.57621100	-0.55626100
C	2.08932200	-1.86735900	-0.58913700
C	3.08112100	0.61107700	0.33684700
C	3.13666900	-1.80548300	0.30778900
H	1.71732200	-2.81746800	-0.95296600
H	3.46742800	1.56075400	0.68969100
C	3.62916000	-0.57791000	0.76907400
H	3.59739100	-2.72352700	0.65042800
H	4.45965800	-0.56891800	1.46329100
C	-1.14733500	1.10394700	1.43511500
C	-0.15753800	0.55030900	2.18442800
C	-1.70078600	0.02888000	0.67054800
H	0.51634400	1.02969000	2.87648400
N	-0.07475900	-0.80445900	1.94983000
C	-1.00064900	-1.14776200	1.00524700
C	-2.68661100	-0.07123100	-0.30288600
H	0.68584300	-1.39176300	2.24160200
C	-1.26911600	-2.37512600	0.40798500
C	-2.96725200	-1.26397200	-0.91957900
H	-0.72055300	-3.26543100	0.68743700
C	-2.25420100	-2.41402100	-0.55491400
H	-3.74531200	-1.30006700	-1.66976400
H	-2.49370000	-3.35276300	-1.03731900
C	-1.53587100	2.53501600	1.37629700
H	-1.38435800	2.93786700	0.37021000
H	-2.58960500	2.68365500	1.61985000
H	-0.93968600	3.12927200	2.06989600
F	-3.35627700	1.02834800	-0.66144300
H	-0.18364200	-1.10791400	-2.15693800
N	0.48275100	-0.41775800	-1.85603100
C	1.19650600	1.59178000	-1.14688000
C	1.33535900	3.05083500	-0.92472900
H	1.23589100	3.29734000	0.13739300
H	2.31304100	3.41970700	-1.24576300
H	0.57334200	3.61161600	-1.46726600

Entry: H-4Cl
E^{CP} **(a.u.):** -1265.54061

Entry: H-4Br
E^{CP} **(a.u.):** -3379.44646

C	0.27918900	1.01166700	-1.83860300
C	1.74953800	-0.48650600	-1.07980900
H	-0.60727500	1.34884900	-2.35367900
C	2.11866500	0.78507500	-0.59696600
C	2.45159200	-1.63319300	-0.73448200
C	3.22119900	0.90175300	0.24754900
C	3.52942000	-1.49256500	0.11686500
H	2.16025400	-2.60414000	-1.11680800
H	3.52070000	1.87329500	0.62425600
C	3.91293800	-0.23665300	0.60434900
H	4.09965200	-2.36793900	0.40146400
H	4.77064300	-0.16384400	1.26064200
C	-0.98465700	0.83872100	1.56123200
C	0.11196100	0.38098500	2.22226400
C	-1.45596300	-0.26394900	0.77519800
H	0.76511700	0.91717500	2.89244400
N	0.34198400	-0.94179800	1.92846900
C	-0.59218500	-1.35656500	1.02208600
C	-2.48077400	-0.46704700	-0.14823700
H	1.20226500	-1.42804300	2.11317000
C	-0.73341800	-2.58888500	0.39925900
C	-2.63404600	-1.67734600	-0.78493100
H	-0.05409900	-3.40301700	0.61828300
C	-1.76085300	-2.73440900	-0.50696100
H	-3.44069400	-1.80394200	-1.49395900
H	-1.90709000	-3.68185500	-1.00908500
C	-1.52559200	2.21973300	1.64522800
H	-1.49650700	2.72171900	0.67505700
H	-2.56383900	2.23885300	1.98072100
H	-0.93557900	2.81375800	2.34519700
H	0.03261900	-1.06458600	-2.15948100
N	0.63883200	-0.31905700	-1.86340500
C	1.16558800	1.72651300	-1.09620100
C	1.14200100	3.17629400	-0.78810400
H	1.03343100	3.34005700	0.28957900
H	2.06466300	3.67471500	-1.09597400
H	0.31017000	3.67725300	-1.28451900
Cl	-3.58280900	0.83034900	-0.54477500

Entry: H-4I
E^{CP} (a.u.): -1101.10396

C	-0.73253300	-0.97522200	-1.80003300
C	-2.49580200	0.24620600	-1.18249100
H	0.22254000	-1.16167100	-2.26638300
C	-2.64575300	-1.05244800	-0.65476200
C	-3.42085900	1.25040600	-0.93186400
C	-3.75221900	-1.34293400	0.14132500
C	-4.50049700	0.94031800	-0.12893200
H	-3.29796100	2.24259400	-1.34921800
H	-3.88347800	-2.33782400	0.55200900
C	-4.66657800	-0.34476900	0.40361200
H	-5.24276500	1.70038200	0.08021800
H	-5.53238400	-0.55398700	1.01874300
C	0.18413300	-0.52021300	1.75812800
C	-1.03054500	-0.23536300	2.29925400
C	0.52997000	0.60941500	0.94076400
H	-1.63894600	-0.85046300	2.94334400
N	-1.45888300	1.00885400	1.90943500
C	-0.53405900	1.53948000	1.05641900
C	1.57937700	0.95514000	0.08286000
H	-2.40349600	1.34285500	1.99372200
C	-0.56367100	2.75007100	0.38057500
C	1.55304400	2.14706500	-0.61144400
H	-1.39266100	3.43501700	0.50750100
C	0.48723600	3.04016100	-0.45959000
H	2.37166800	2.40202800	-1.27151700
H	0.50309900	3.97516800	-1.00466700
C	0.93148700	-1.78345200	1.98961000
H	1.11099500	-2.32385800	1.05737300
H	1.90675700	-1.61388600	2.44965300

C	-0.46819600	-0.96244600	-1.85255900
C	-2.13706300	0.34909400	-1.16267300
H	0.46206800	-1.19621400	-2.34698700
C	-2.34831300	-0.94708900	-0.65081500
C	-2.99095700	1.40308300	-0.86752700
C	-3.44350500	-1.18226400	0.17915200
C	-4.06037100	1.14633600	-0.03336000
H	-2.81645200	2.39400400	-1.26937800
H	-3.62108500	-2.17357600	0.58108500
C	-4.28636100	-0.13517600	0.48640700
H	-4.74649400	1.94671100	0.21366600
H	-5.14157100	-0.30135700	1.12892100
C	0.61074700	-0.70317900	1.67830100
C	-0.57452900	-0.41439500	2.27941400
C	0.95489300	0.45779300	0.90877400
H	-1.17449400	-1.04404200	2.91738000
N	-0.98349700	0.85791100	1.96485800
C	-0.07463600	1.40953400	1.10801200
C	1.97812800	0.81527700	0.02900500
H	-1.90837700	1.21882100	2.12113500
C	-0.08672200	2.65424000	0.49448300
C	1.97598400	2.03864200	-0.60215700
H	-0.88898700	3.35674900	0.68161100
C	0.94635600	2.95520400	-0.36444300
H	2.78245300	2.29100300	-1.27726800
H	0.97471000	3.91711300	-0.85973900
C	1.33849900	-1.99199800	1.80907900
H	1.48195700	-2.47518300	0.84016800
H	2.32942000	-1.86697800	2.24957500
H	0.77406100	-2.67733000	2.44392200
H	-0.49415500	1.11815800	-2.23965700
N	-0.99579300	0.30905600	-1.91787900
C	-1.26968600	-1.76821500	-1.10620900
C	-1.06790800	-3.19831800	-0.77240100
H	-1.00385900	-3.34021600	0.31149200
H	-1.89341000	-3.82114000	-1.12660000
H	-0.14630200	-3.58326200	-1.21055000
Br	3.39735400	-0.38539300	-0.36715800

Entry: H-5F
E^{CP} (a.u.): -905.18109

C	0.55767400	0.79563000	-2.02576200
C	1.36890900	-0.99001700	-0.96057300
H	-0.12798500	1.34039500	-2.65561800
C	2.16970600	0.12407300	-0.63682400
C	1.60858300	-2.24717600	-0.42337500
C	3.22831400	-0.03383000	0.25587700
C	2.65618400	-2.37416100	0.46657200
H	0.98992400	-3.09638300	-0.68744600
H	3.85463900	0.81219300	0.51605700
C	3.45937400	-1.27729200	0.80570800
H	2.87007900	-3.34231400	0.90141200
H	4.27834200	-1.41658900	1.49968800
C	-0.79518600	1.69991500	1.26674500
C	0.19135800	1.10076000	1.98772100
C	-1.60161200	0.64400500	0.74313000
H	1.01600400	1.55338800	2.51611000
N	0.03793500	-0.26532200	1.97108000
C	-1.04330400	-0.57214700	1.18577100
C	-2.72548700	0.62805500	-0.08418200
H	0.74449800	-0.92303100	2.25167600
C	-1.57829000	-1.79840800	0.82158100
C	-3.23039000	-0.59620100	-0.44063700
H	-1.14323100	-2.72662800	1.17056000
C	-2.68099700	-1.80277800	-0.00879400
H	-3.14619000	-2.72700000	-0.32415600
C	-0.99054300	3.14680600	1.01048100
H	-0.86791900	3.37358900	-0.05411700
H	-1.99209200	3.47912300	1.29502600
H	-0.27012100	3.75101100	1.56260200

H	0.36030000	-2.43805600	2.65038900
H	-0.86597600	1.09219100	-2.22460200
N	-1.33159700	0.26138700	-1.90330300
C	-1.50622700	-1.81454800	-1.06166200
C	-1.23094600	-3.22496100	-0.69764000
H	-1.22147600	-3.35133000	0.38995800
H	-1.99097200	-3.90554300	-1.09026300
H	-0.26203400	-3.55196700	-1.07732700
I	3.23918500	-0.30376300	-0.23672900

Entry: H-5Cl
E^{CP} (a.u.): -1265.54015

C	-0.69530800	0.73547400	2.05827300
C	-1.45141800	-1.08078000	1.00507300
H	-0.00770600	1.31477400	2.65444200
C	-2.35105500	-0.02127800	0.76849100
C	-1.63726000	-2.33973900	0.45097100
C	-3.45532300	-0.23657400	-0.05445400
C	-2.73098300	-2.52272100	-0.37061600
H	-0.94317000	-3.14712100	0.65032800
H	-4.15944300	0.56540300	-0.24629400
C	-3.63217700	-1.47988200	-0.62431700
H	-2.90480500	-3.49384000	-0.81658500
H	-4.48564600	-1.66342800	-1.26440400
C	0.29580900	1.83720000	-1.30744000
C	-0.72013600	1.22090900	-1.96922600
C	1.20309200	0.80226800	-0.92344500
H	-1.61454300	1.65020600	-2.39335200
N	-0.49103900	-0.13364500	-2.04851800
C	0.67370400	-0.41814000	-1.38862000
C	2.39546500	0.80598800	-0.20077800
H	-1.18186200	-0.81585200	-2.30938000
C	1.30676200	-1.62870700	-1.15025200
C	3.00456400	-0.40343600	0.03756500
H	0.89103600	-2.56044700	-1.51290200
C	2.48001900	-1.61390000	-0.42481200
H	3.01196300	-2.53176200	-0.21509600
C	0.43953700	3.27714700	-0.98716900
H	0.43470700	3.43589400	0.09600500
H	1.37924900	3.68657400	-1.36627800
H	-0.37413300	3.86474000	-1.41302800
H	0.40448200	-1.07533400	2.00935400
N	-0.45756800	-0.59929200	1.81317600
C	-1.84899300	1.12783400	1.45562100
C	-2.46778100	2.47466600	1.45845100
H	-2.55133000	2.86856600	0.44054100
H	-3.47788600	2.45621000	1.87569200
H	-1.87983700	3.18373400	2.04206300
H	2.82940700	1.72591400	0.17088300
Cl	4.49521400	-0.44421500	0.95561800

Entry: H-5I
E^{CP} (a.u.): -1101.10424

C	-1.34097900	0.46975700	2.06464600
C	-2.28948200	-1.21273700	0.94702800
H	-0.55680200	0.97428000	2.60683500
C	-3.19133600	-0.12916900	0.97117600
C	-2.58322300	-2.39824200	0.28763100
C	-4.41330800	-0.24571400	0.31065900
C	-3.79446000	-2.48288000	-0.36940600
H	-1.88192300	-3.22398400	0.28209500
H	-5.11990200	0.57663300	0.31937100
C	-4.70276800	-1.41570600	-0.35965200
H	-4.05406400	-3.39488600	-0.89188500
H	-5.64801900	-1.52166000	-0.87649300
C	-0.89463400	1.96876100	-1.32943400
C	-1.96617900	1.33354600	-1.87398100
C	0.09686800	0.96087000	-1.11920000
H	-2.92549500	1.73878800	-2.15578300
N	-1.69624000	-0.00650000	-2.04565100

H	-0.39133300	-1.09907800	-2.12029000
N	0.40203800	-0.55857400	-1.82678200
C	1.63120000	1.25358100	-1.32852300
C	2.14089200	2.64273000	-1.24251700
H	2.08466300	3.01575800	-0.21457200
H	3.18813100	2.71014700	-1.54790100
H	1.56527400	3.32042000	-1.87357100
H	-3.19044600	1.53704700	-0.44502000
F	-4.30448300	-0.65067000	-1.23943500

Entry: H-5Br
E^{CP} (a.u.): -3379.44645

C	0.96763600	0.56818000	-2.05965800
C	1.86948700	-1.15623100	-0.96643600
H	0.20644200	1.09254700	-2.61563200
C	2.77140900	-0.07412200	-0.91479000
C	2.13998100	-2.36681200	-0.34318300
C	3.96855700	-0.21592800	-0.21494700
C	3.32665800	-2.47700800	0.35383100
H	1.43990700	-3.19187700	-0.39539000
H	4.67376700	0.60631500	-0.16747700
C	4.23449500	-1.41145300	0.41901500
H	3.56781700	-3.40931700	0.84868100
H	5.16030300	-1.53881200	0.96528700
C	0.36870700	1.90073700	1.36157800
C	1.42083800	1.26093400	1.93803100
C	-0.60164600	0.89050300	1.07928700
H	2.36372700	1.66586100	2.27104600
N	1.15604300	-0.08511100	2.06394100
C	-0.07044500	-0.34007400	1.51328900
C	-1.85198900	0.92434700	0.46419800
H	1.85334200	-0.78757100	2.24235400
C	-0.76119400	-1.53218300	1.35105400
C	-2.51718700	-0.26739500	0.29253200
H	-0.34682900	-2.47261600	1.69285100
C	-1.99129000	-1.48817500	0.72698600
H	-2.56317500	-2.39443700	0.58007700
C	0.23310700	3.34055700	1.03718400
H	0.11332900	3.48584500	-0.04120100
H	-0.64318300	3.78360200	1.51720900
H	1.10863500	3.90762000	1.35400200
H	-0.07816600	-1.25389400	-1.77902900
N	0.78290800	-0.74543600	-1.68908300
C	2.17304300	1.01696100	-1.61833500
C	2.76178800	2.36817800	-1.77702000
H	2.93421900	2.83557400	-0.80171300
H	3.72803800	2.33273500	-2.28671600
H	2.10683000	3.02571900	-2.34931800
H	-2.28334700	1.85663400	0.12124000
Br	-4.21617300	-0.26789200	-0.56102800

Entry: H-6F
E^{CP} (a.u.): -905.18137

C	-0.78201200	0.62841100	2.10845100
C	-1.14337300	-1.20964700	0.89461300
H	-0.26630900	1.25334900	2.82052100
C	-2.12204900	-0.25274000	0.55779100
C	-1.09117700	-2.45402100	0.28141900
C	-3.06461700	-0.55690600	-0.42278100
C	-2.03025100	-2.72605000	-0.69330100
H	-0.33725400	-3.18282700	0.55342600
H	-3.82395400	0.16846900	-0.69302700
C	-3.00830400	-1.78609500	-1.04557800
H	-2.01943900	-3.68949900	-1.18718300
H	-3.73429600	-2.03917100	-1.80748300
C	0.39408500	1.95049500	-1.14910300
C	-0.35325100	1.11928100	-1.91913300
C	1.43637000	1.14600400	-0.58680600
H	-1.24607900	1.33191400	-2.48584400
N	0.17656300	-0.15537400	-1.90281200

C	-0.44349800	-0.26351500	-1.56020900
C	1.37540400	0.99017300	-0.56496600
H	-2.39373300	-0.71091500	-2.21588900
C	0.26425900	-1.45255400	-1.46086700
C	2.06533000	-0.19764500	-0.45635200
H	-0.15845500	-2.38836000	-1.80574500
C	1.52359800	-1.41163800	-0.89766800
H	2.10305600	-2.32076100	-0.80591700
C	-0.76042700	3.40332000	-0.98085800
H	-0.56413400	3.53050500	0.08838900
H	0.06844900	3.87589400	-1.51402700
H	-1.66830800	3.95811700	-1.21917400
H	-0.31042700	-1.34035300	1.67298800
N	-1.17442100	-0.83010600	1.64129800
C	-2.56268100	0.93447000	1.69048500
C	-3.13865100	2.27960700	1.92850000
H	-3.40512600	2.76219800	0.98297600
H	-4.05054000	2.23332300	2.52945000
H	-2.43245900	2.92991300	2.44568600
H	1.80799000	1.92199500	-0.22134700
I	3.99663300	-0.19865500	0.38195800

Entry: H-6Cl
E^{CP} (a.u.): -1265.54033

C	-0.88994100	0.12551000	2.20887200
C	-0.78786200	-1.59927900	0.79711300
H	-0.53796700	0.78527600	2.98618800
C	-1.99727700	-0.91050800	0.57236600
C	-0.41874300	-2.70323700	0.04078900
C	-2.85655600	-1.35212100	-0.43246400
C	-1.27945700	-3.11008100	-0.95806800
H	0.51882200	-3.21656400	0.22115300
H	-3.79377900	-0.83762900	-0.61436100
C	-2.48981600	-2.44241500	-1.19308200
H	-1.02161100	-3.96848800	-1.56510600
H	-3.14699400	-2.80067800	-1.97530000
C	-0.50763300	2.41896300	-0.90029600
C	-1.14239800	1.56606900	-1.74703400
C	0.70940500	1.77091600	-0.52053400
H	-2.10069100	1.67463000	-2.23065200
N	-0.38363200	0.43292100	-1.94643600
C	0.74095500	0.52320500	-1.17618300
C	1.74809600	2.10988200	0.34438500
H	-0.72863400	-0.42027600	-2.35197300
C	1.76218200	-0.39322400	-0.97520500
C	2.76444300	1.20569100	0.56621900
H	1.77547000	-1.35870000	-1.46268100
C	2.74721300	-0.02927200	-0.08707100
C	-0.98561700	3.73342000	-0.40870800
H	-1.09071300	3.72764500	0.68099300
H	-0.29177900	4.54044200	-0.65710200
H	-1.95697000	3.98756000	-0.83426300
C	-3.11607800	1.20426500	1.57848600
H	-3.17984600	1.78822700	0.65450600
H	-4.09536600	0.74616700	1.73853200
H	-2.93662800	1.90165900	2.39742700
C	-2.04068300	0.18794500	1.48675200
N	-0.13701800	-0.95802900	1.81490500
H	0.80242300	-1.15126300	2.11143400
H	1.75127300	3.06710700	0.85249700
H	3.57815900	1.43218700	1.24118200
Cl	4.01988100	-1.17976000	0.26766000

Entry: H-6I
E^{CP} (a.u.): -1101.10504

C	-1.37917800	-0.12035200	2.20729800
C	-1.18918400	-1.74802000	0.69301500
H	-1.03452500	0.56354500	2.96670500
C	-2.51625500	-1.27128500	0.67163600
C	-0.74696500	-2.73405200	-0.17846400

C	1.25585000	-0.16784400	-1.06597900
C	2.47947000	1.41176200	0.29673000
H	-0.31194500	-0.97805400	-2.21205300
C	2.08285900	-1.21547700	-0.68068000
C	3.30684600	0.38255800	0.69478800
H	1.96325900	-2.22782900	-1.04296700
C	3.09089600	-0.90128400	0.19841400
C	0.16981900	3.39065000	-0.88024900
H	-0.01449800	3.56427100	0.18511500
H	1.03547300	3.99771100	-1.15760000
H	-0.69169300	3.76904800	-1.43165700
C	-2.63792800	2.18023400	1.28450200
H	-2.56976700	2.62907100	0.28776100
H	-3.69989400	2.02387900	1.49025700
H	-2.26273000	2.91042400	2.00238900
C	-1.87241100	0.91253900	1.34756400
N	-0.34583800	-0.65377200	1.85756500
H	0.51872100	-1.04384100	2.18590700
H	2.63532000	2.41559500	0.67428000
H	4.13032000	0.54212900	1.37739100
F	3.91275100	-1.87687000	0.60372600

Entry: H-6Br
E^{CP} (a.u.): -3379.44688

C	-0.99359500	-0.13016400	2.20126800
C	-0.94720500	-1.77415100	0.69442400
H	-0.59309000	0.51883500	2.96375300
C	-2.21804700	-1.16448000	0.65014300
C	-0.59403900	-2.80403700	-0.16670800
C	-3.15744800	-1.61783200	-0.27503500
C	-1.53616100	-3.22107000	-1.08536700
H	0.38936300	-3.25786200	-0.12334000
H	-4.14287100	-1.16740700	-0.31935200
C	-2.80884600	-2.63567000	-1.13788800
H	-1.29297000	-4.02305200	-1.77040400
H	-3.52751200	-2.99978800	-1.86102500
C	-1.38766100	2.43864800	-0.83374300
C	-1.93889700	1.48917100	-1.63608200
C	-0.06864100	1.98121400	-0.52448200
H	-2.92900900	1.45019000	-2.06297300
N	-1.03360400	0.48000700	-1.87965400
C	0.10909500	0.74519400	-1.17849900
C	0.95831400	2.47984000	0.27457400
H	-1.27422000	-0.42222900	-2.25478200
C	1.26596400	-0.00685800	-1.03926000
C	2.10955300	1.73844500	0.43603800
H	1.39027000	-0.96177000	-1.53250200
C	2.23923400	0.50855000	-0.21453100
C	-2.01575300	3.68701900	-0.33792800
H	-2.01113300	3.72302400	0.75561700
H	-1.48578800	4.57706100	-0.68666000
H	-3.05225900	3.76774100	-0.66736000
C	-3.34506000	0.81577300	1.89430400
H	-3.60736500	1.38674200	0.99719900
H	-4.24591200	0.28513000	2.21327900
H	-3.08560200	1.52982100	2.67674500
C	-2.22349000	-0.11494700	1.62078800
N	-0.22558700	-1.14115800	1.66898200
H	0.75642600	-1.26992100	1.83416200
H	0.85014800	3.43298500	0.77897100
H	2.91583200	2.09549100	1.06220100
Br	3.80217500	-0.52928000	0.09768800

Entry: H-7F
E^{CP} (a.u.): -905.18041

C	0.89277500	-1.10789900	1.95535300
C	1.18856200	0.90997800	1.04643900
H	0.42973200	-1.84167000	2.59636000
C	2.09928100	0.01348900	0.45126700
C	1.11051700	2.24061800	0.65935300

C	-3.42028100	-1.81374000	-0.23998000
C	-1.65844700	-3.24314000	-1.08152700
H	0.27946100	-3.08209300	-0.15535200
H	-4.44618400	-1.46338500	-0.26635200
C	-2.98499800	-2.79046100	-1.11107700
H	-1.34690000	-4.01523300	-1.77340500
H	-3.67468300	-3.22434400	-1.82385200
C	-2.01269000	2.31816000	-0.84697800
C	-2.47081000	1.34007800	-1.67300100
C	-0.65351800	1.98670100	-0.55428000
H	-3.45510200	1.21238500	-2.09581600
N	-1.47093900	0.43262000	-1.94557400
C	-0.35702700	0.79099700	-1.23867000
C	0.31984100	2.56399100	0.25801600
H	-1.62367000	-0.48049800	-2.33919500
C	0.86637200	0.15009000	-1.11137500
C	1.53711200	1.93349500	0.40583200
H	1.06994000	-0.78264600	-1.62216000
C	1.78914800	0.73351500	-0.27018800
C	-2.76336600	3.47384600	-0.30040700
H	-2.79684400	3.43486100	0.79310500
H	-2.30202300	4.42656100	-0.57209400
H	-3.79207000	3.48693800	-0.66219100
C	-3.82380100	0.57286200	1.94333000
H	-4.17100400	1.10095600	1.04951800
H	-4.65213400	-0.05223800	2.28695300
H	-3.62818700	1.31867500	2.71456900
C	-2.61332900	-0.22986900	1.64611800
N	-0.52053900	-1.04529400	1.65742600
H	0.47007700	-1.08223700	1.81737700
H	0.11742600	3.48873400	0.78623600
H	2.29835800	2.35713100	1.04690600
I	3.60552200	-0.27160900	0.08585400

Entry: H-7Cl
***E*^CP (a.u.):** -1265.54167

C	1.71287600	-0.85508200	1.84147900
C	0.95156900	1.12048200	1.13370000
H	1.70859700	-1.76101800	2.42727500
C	2.10977900	0.79870500	0.39731900
C	0.23346300	2.28430500	0.89991000
C	2.54616100	1.66669600	-0.60166100
C	0.68049200	3.12099400	-0.10224900
H	-0.65663400	2.52069000	1.47018300
H	3.43566400	1.43339500	-1.17645000
C	1.82626900	2.81677700	-0.84857900
H	0.13600600	4.03323600	-0.31178300
H	2.15261400	3.50273000	-1.61990300
C	0.21849200	-1.87728700	-1.31649000
C	0.37527100	-0.71805600	-2.00917400
C	-0.90299700	-1.67833000	-0.45114300
H	1.12731100	-0.45590600	-2.73659000
N	-0.60376000	0.18699100	-1.66354200
C	-1.38051300	-0.37358700	-0.69322800
C	-1.52774300	-2.47401500	0.50768000
H	-0.58192300	1.17133100	-1.87083300
C	-2.47376800	0.11803600	0.00190000
C	-2.60037600	-1.96103500	1.20564900
C	-3.07813900	-0.66947100	0.95565700
C	1.03494200	-3.11164100	-1.40597500
H	1.45094400	-3.38336100	-0.43111600
H	0.44218900	-3.96524700	-1.74536300
H	1.86635900	-2.98961400	-2.10108200
H	-0.07781300	-0.00520100	2.59576500
N	0.73805900	0.10048700	2.02147300
C	2.58020600	-0.46562600	0.86937800
C	3.75049800	-1.20299900	0.33741200
H	3.60785200	-1.44359400	-0.72157000
H	4.66912500	-0.61544400	0.40980100
H	3.91115600	-2.14069900	0.87053800
H	-1.17283900	-3.47984300	0.69919700

C	2.94510100	0.46874900	-0.55845500
C	1.95135900	2.66306400	-0.35066200
H	0.40136700	2.91981200	1.11648400
H	3.65142300	-0.20870300	-1.02521000
C	2.86155200	1.78648000	-0.95557500
H	1.91285800	3.69501600	-0.67541800
H	3.51236000	2.15720000	-1.73722300
C	-0.74972400	-1.74318000	-1.27848400
C	-0.06223800	-0.84127300	-2.02964800
C	-1.65578900	-0.98745400	-0.46993500
H	0.72907400	-1.01184000	-2.74257200
N	-0.50313000	0.43781300	-1.77159800
C	-1.45691000	0.36613900	-0.79676200
C	-2.58615800	-1.33400400	0.51218900
H	-0.01332100	1.27969300	-2.02251000
C	-2.17761500	1.35402500	-0.14844500
C	-3.28134700	-0.33257400	1.15349600
C	-3.07914600	1.01663700	0.82886600
C	-0.59344500	-3.21706800	-1.25324200
H	-0.39603300	-3.57068900	-0.23677400
H	-1.49588400	-3.72944300	-1.59700800
H	0.23414700	-3.54147200	-1.88512800
H	-0.34855600	0.53615600	2.44714500
N	0.47556600	0.20482700	1.97897700
C	1.89214100	-1.26860400	1.04778100
C	2.59973900	-2.51676900	0.67558300
H	2.41339700	-2.76717800	-0.37476100
H	3.68207500	-2.42367400	0.79525700
H	2.26818400	-3.36141000	1.28044800
H	-2.75288800	-2.37386200	0.76653200
H	-4.00751700	-0.58066400	1.91646800
F	-1.97599500	2.63324300	-0.48107500
H	-3.63544300	1.80292800	1.32122600

Entry: H-7Br
***E*^CP (a.u.):** -3379.44843

C	-2.37347100	0.53496000	1.72015800
C	-1.04002600	-1.16331800	1.15416200
H	-2.65425100	1.43730800	2.24110800
C	-2.22650600	-1.25811200	0.39910200
C	0.00062500	-2.06874000	1.00458000
C	-2.36427100	-2.29034000	-0.52728300
C	-0.15750300	-3.07535400	0.07447500
H	0.90949400	-1.98152800	1.58759300
H	-3.27115200	-2.37985900	-1.11485900
C	-1.32913900	-3.18788200	-0.68486900
H	0.63876100	-3.79547100	-0.06680300
H	-1.42143000	-3.99677500	-1.39838500
C	-1.05411900	1.70669900	-1.42657000
C	-0.74513400	0.58396500	-2.12897100
C	-0.00073300	1.88697200	-0.47738700
H	-1.30203600	0.09550000	-2.91276900
N	0.45204800	0.06077000	-1.69446400
C	0.91373300	0.82942400	-0.66876300
C	0.23066400	2.82308200	0.52810000
H	0.82761800	-0.83553800	-1.95023700
C	2.04458000	0.71907300	0.12500600
C	1.35073400	2.69073300	1.32115500
C	2.25909000	1.64334300	1.12347400
C	-2.24977100	2.57210000	-1.56397700
H	-2.80513100	2.62103400	-0.62171200
H	-1.98126800	3.59734100	-1.83174000
H	-2.92834200	2.19537000	-2.33009700
H	-0.42495900	0.32121900	2.52634400
N	-1.16328100	-0.07132300	1.97113800
C	-3.06180800	-0.16057200	0.77562300
C	-4.39038800	0.15403200	0.19847500
H	-4.31992000	0.29452200	-0.88516500
H	-5.11146400	-0.64940100	0.36903500
H	-4.80405400	1.06953600	0.62376100
H	-0.46567300	3.63911500	0.68285100

H	-3.09918700	-2.56409900	1.95305100
H	-3.93285300	-0.28374300	1.49454800
Cl	-3.07444500	1.71652100	-0.35533500

Entry: H-7I
E^{CP} (a.u.): -1101.10660

C	-2.77231700	0.32125600	1.71476500
C	-1.27975400	-1.24188200	1.15784600
H	-3.14209500	1.18992200	2.23707900
C	-2.44197000	-1.44039700	0.38511100
C	-0.15767600	-2.04531600	1.01462900
C	-2.47051000	-2.47318200	-0.55084800
C	-0.20798100	-3.05274700	0.07326600
H	0.73182300	-1.88162200	1.61131100
H	-3.35730500	-2.64227500	-1.15126700
C	-1.35377100	-3.26789400	-0.70269700
H	0.65612600	-3.69093700	-0.06397500
H	-1.35995900	-4.07475100	-1.42436200
C	-1.55767400	1.70754700	-1.42070900
C	-1.14402100	0.61125300	-2.11055300
C	-0.54570800	1.97113000	-0.44640400
H	-1.63936600	0.08179100	-2.90885200
N	0.08046900	0.18682400	-1.64638500
C	0.46063800	0.99381100	-0.61527700
C	-0.41701200	2.92475300	0.56007600
H	0.53467900	-0.67538300	-1.89369500
C	1.58916600	0.98602300	0.19332200
C	0.69216800	2.88898100	1.37756400
C	1.69460000	1.92890300	1.19567300
C	-2.79669200	2.49611100	-1.62557500
H	-3.36120800	2.60738700	-0.69544100
H	-2.57747800	3.50638500	-1.98183500
H	-3.45164700	2.02397300	-2.35851800
H	-0.82114000	0.28762900	2.54190900
N	-1.51375400	-0.17085800	1.97833400
C	-3.38044800	-0.42792000	0.75666000
C	-4.72130500	-0.22979900	0.15578100
H	-4.63827500	0.01081600	-0.90971800
H	-5.33900600	-1.12785200	0.23400200
H	-5.25903300	0.58774000	0.63744500
H	-1.18269700	3.67963400	0.69644300
H	0.81082500	3.62262100	2.16451500
H	2.56889400	1.93772100	1.83339600
I	3.12682000	-0.40487500	-0.14279000

Entry: 4Cl-4Cl
E^{CP} (a.u.): -1725.14575

C	-0.04602900	-0.68002900	2.05850400
C	1.02620900	1.09749900	1.24867600
H	-0.83392800	-1.20536300	2.57544100
C	1.72345600	-0.06622600	0.84869300
C	1.41775000	2.37824500	0.88341100
C	2.84490300	0.11800400	0.04030800
C	2.53279800	2.50255800	0.08564300
H	0.85337500	3.24389000	1.20649500
C	3.24731000	1.37678300	-0.34022600
H	2.87311100	3.48430700	-0.21692400
H	4.12439900	1.48634500	-0.96314900
C	-1.01871100	-1.19367500	-1.38389000
C	0.04597600	-0.68086800	-2.05826600
C	-1.72341500	-0.06647400	-0.84860500
H	0.83377900	-1.20645200	-2.57509300
N	0.03931000	0.69179300	-1.99957900
C	-1.02608800	1.09704600	-1.24902400
C	-2.84488400	0.11814300	-0.04033800
H	0.78228200	1.29060300	-2.31060800
C	-1.41746300	2.37794500	-0.88414500
C	-3.24714000	1.37709000	0.33980800
H	-0.85298600	3.24341900	-1.20750500
C	-2.53247400	2.50264400	-0.08638000

H	1.54890400	3.41013100	2.10500800
H	3.14216000	1.56184000	1.74298200
Br	3.27320100	-0.69059600	-0.18235900

Entry: 4F-4F
E^{CP} (a.u.): -1004.42800

C	-0.16322000	0.75130000	2.07894600
C	-1.20126200	-0.97316400	1.11173900
H	0.58235100	1.23648300	2.68933600
C	-1.82496500	0.22137800	0.69732300
C	-1.60041000	-2.22599700	0.65765800
C	-2.86344200	0.11007700	-0.21971800
C	-2.63884600	-2.27557000	-0.24547100
H	-1.10391900	-3.12610400	0.99687200
C	-3.27397000	-1.10937100	-0.69429700
H	-2.98088400	-3.23302400	-0.61634400
H	-4.09228900	-1.15165200	-1.40010300
C	1.14904400	1.31366000	-1.32784700
C	0.16341200	0.75284600	-2.07829800
C	1.82497200	0.22140100	-0.69705100
H	-0.58182200	1.23872100	-2.68855100
N	0.19741000	-0.61927500	-1.96954500
C	1.20053500	-0.97262600	-1.11189900
C	2.86366400	0.10906600	0.21964000
H	-0.51462900	-1.24185900	-2.30584600
C	1.59917200	-2.22591000	-0.65860900
C	3.27368700	-1.11084400	0.69344500
H	1.10213400	-3.12558000	-0.99817300
C	2.63785200	-2.27649000	0.24418900
H	4.09217100	-1.15391900	1.39901300
H	2.97951100	-3.23431400	0.61445600
C	1.43740800	2.76087400	-1.17005500
H	1.33942000	3.07182300	-0.12662400
H	2.45442800	3.01063200	-1.47875700
H	0.74700000	3.36078000	-1.76439700
F	3.46152200	1.21958600	0.66137900
H	0.51412900	-1.24380800	2.30485100
N	-0.19818700	-0.62080300	1.96987400
C	-1.14843000	1.31298200	1.32861000
C	-1.43600300	2.76037100	1.17107600
H	-1.33965300	3.07078000	0.12733200
H	-2.45231000	3.01095700	1.48146400
H	-0.74407800	3.35989500	1.76403600
F	-3.46055600	1.22117300	-0.66106200

Entry: 4Br-4Br
E^{CP} (a.u.): -5952.95730

C	-0.22878500	-0.58125900	2.11419400
C	0.81012700	1.26344900	1.42542300
H	-1.01284800	-1.15847800	2.57850800
C	1.58078900	0.14439400	1.02781700
C	1.16925700	2.57298300	1.13927600
C	2.74165900	0.40968400	0.29867200
C	2.32409200	2.77818200	0.42033400
H	0.54765100	3.40012100	1.45824900
C	3.10997700	1.70085200	-0.00358300
H	2.63942800	3.78519100	0.18012300
H	4.01893700	1.87838300	-0.56255000
C	-0.89334600	-1.02861000	-1.48716100
C	0.22845600	-0.58252600	-2.11419400
C	-1.58082600	0.14405300	-1.02796900
H	1.01230800	-1.16014100	-2.57837900
N	0.27986900	0.78973900	-2.09971900
C	-0.80970000	1.26272400	-1.42572800
C	-2.74159100	0.40989000	-0.29888900
H	1.08742100	1.33901900	-2.33463900
C	-1.16829100	2.57243700	-1.13972100
C	-3.10936200	1.70123100	0.00326800
H	-0.54635100	3.39928700	-1.45879300
C	-2.32300600	2.77818400	-0.42074500

H	-4.12424600	1.48695800	0.96265300
H	-2.87265000	3.48452900	0.21589700
C	-1.33265500	-2.63743700	-1.23084100
H	-1.32656600	-2.93907400	-0.18024000
H	-2.31962600	-2.88734300	-1.62400200
H	-0.59565500	-3.24406800	-1.75930300
H	-0.78195300	1.29164800	2.31055000
N	-0.03929400	0.69259200	1.99923700
C	1.01865500	-1.19317100	1.38437100
C	1.33243000	-2.63701800	1.23178600
H	1.32582900	-2.93913800	0.18133100
H	2.31957100	-2.88681100	1.62458300
H	0.59562200	-3.24337000	1.76083500
Cl	-3.75244300	-1.26145900	0.53446400
Cl	3.75219300	-1.26190100	-0.53419700

Entry: 4I-4I
E^{CP} (a.u.): -1396.27270

C	0.39313900	0.15443400	-2.02202800
C	-0.95281200	1.76255500	-1.28197000
H	1.29142500	-0.26252500	-2.45098000
C	-1.57439200	0.51421600	-1.02559000
C	-1.50847300	2.97890100	-0.91423300
C	-2.80372200	0.54690200	-0.35859900
C	-2.71937300	2.95813400	-0.25928900
H	-1.00121900	3.90968300	-1.13647900
C	-3.36446400	1.74911200	0.01922300
H	-3.18995600	3.88612200	0.03913300
H	-4.31685600	1.75900000	0.53226900
C	0.76395500	-0.52701100	1.63461200
C	-0.31001100	0.11037300	2.17473500
C	1.59289400	0.50030500	1.06869500
H	-1.16755700	-0.32077400	2.66747000
N	-0.19916300	1.46785600	2.01388300
C	0.95155100	1.73771000	1.33214300
C	2.78664100	0.55188100	0.34117000
H	-0.92571300	2.13574700	2.19766900
C	1.46770700	2.96277600	0.93644500
C	3.30433600	1.76210400	-0.07152400
H	0.95125000	3.88496400	1.17247800
C	2.65060300	2.96106600	0.23214600
H	4.22963400	1.78673800	-0.63164400
H	3.09133700	3.89605600	-0.08918100
C	0.97506700	-1.99757000	1.67510800
H	1.09184800	-2.42577100	0.67809700
H	1.86909600	-2.26938300	2.24041000
H	0.11844000	-2.48242400	2.14766200
H	0.97360600	2.18446800	-2.01211100
N	0.23181400	1.51284700	-1.91453400
C	-0.68638500	-0.50139200	-1.51871100
C	-0.83156600	-1.97994700	-1.48445500
H	-0.94420200	-2.35076100	-0.46269900
H	-1.70133400	-2.32718700	-2.04529800
H	0.05694900	-2.44894900	-1.91213700
I	-3.85583800	-1.20694100	0.15670400
I	3.82822500	-1.18715800	-0.24156000

Entry: 4Cl-5Cl
E^{CP} (a.u.): -1725.14546

C	-0.52492400	1.05717900	-1.91982900
C	1.05508700	-0.43023300	-1.40942800
H	-1.47255800	1.39484000	-2.30999900
C	1.46458900	0.83410800	-0.93956900
C	1.82556500	-1.56752300	-1.21559500
C	2.67482000	0.95693200	-0.25764700
C	3.01462300	-1.43525000	-0.52924700
H	1.50444300	-2.53390300	-1.58399700
H	3.02055300	1.91316400	0.11470500
C	3.41993500	-0.18153200	-0.06210600
H	3.65080300	-2.29202800	-0.35554500

H	-4.01821200	1.87919600	0.56227400
H	-2.63791300	3.78534700	-0.18061400
C	-1.26217300	-2.45831400	-1.32058700
H	-1.29463800	-2.74617200	-0.26640700
H	-2.24231000	-2.68569100	-1.74263400
H	-0.52725300	-3.09439200	-1.81676400
H	-1.08710100	1.34066600	2.33402200
N	-0.27971600	0.79102200	2.09936900
C	0.89294700	-1.02791000	1.48743900
C	1.26133700	-2.45782100	1.32157800
H	1.29430400	-2.74615200	0.26755000
H	2.24111900	-2.68544200	1.74431500
H	0.52587900	-3.09343200	1.81757000
Br	3.84535800	-1.00193600	-0.33690200
Br	-3.84566800	-1.00127500	0.33701900

Entry: 4F-5F
E^{CP} (a.u.): -1004.42756

C	0.20363600	0.96175800	1.98496200
C	-1.22489000	-0.56880900	1.21890300
H	1.08934500	1.32285800	2.48491100
C	-1.66791100	0.70229100	0.80105100
C	-1.89356800	-1.73055300	0.86311000
C	-2.81492100	0.80893400	0.01279800
C	-3.01700000	-1.61412000	0.06953700
H	-1.54893200	-2.70143600	1.19642900
H	-3.19754800	1.76323100	-0.32700700
C	-3.45344200	-0.35357600	-0.33526300
H	-3.58560600	-2.48253600	-0.23450500
C	1.35665900	1.07575000	-1.48179500
C	0.26395700	0.60834100	-2.14227000
C	1.88309200	-0.04452300	-0.76235000
H	-0.42052000	1.14436200	-2.78033600
N	0.08880900	-0.73423800	-1.89479700
C	1.06123000	-1.15788800	-1.03456600
C	2.93716000	-0.23751900	0.12318100
H	-0.71667200	-1.27216100	-2.15804000
C	1.27945900	-2.41058600	-0.47091400
C	3.16968700	-1.45664100	0.70781100
H	0.63333300	-3.24752600	-0.70353200
C	2.33770000	-2.54193300	0.40104500
H	4.00417500	-1.56353000	1.38731800
H	2.54110000	-3.50266200	0.85587600
C	1.86998200	2.46844400	-1.48968900
H	1.85673900	2.90538100	-0.48769500
H	2.90073000	2.52354000	-1.84484900
H	1.25880900	3.10027000	-2.13574000
F	3.72720200	0.79586100	0.42851700
N	-0.09287600	-0.38088800	1.97032100
H	0.55269900	-1.10871400	2.22334500
C	-0.73994400	1.66509400	1.30151800
C	-0.79633800	3.12812100	1.06963300
H	-0.77524000	3.35610400	-0.00100100
H	-1.71180700	3.57025900	1.47132800
H	0.04966000	3.63703700	1.53283500
F	-4.55265700	-0.29076200	-1.09831900

Entry: 4Br-5Br
E^{CP} (a.u.): -5952.95744

C	-0.82869400	-1.05281900	2.01758800
C	0.83398700	0.38237100	1.63418400
H	-1.80877500	-1.36122400	2.34724000
C	1.21484600	-0.88270400	1.14356000
C	1.65271400	1.49458000	1.50839800
C	2.44098600	-1.02761200	0.49572700
C	2.86015700	1.33988000	0.85973300
H	1.35148300	2.46034400	1.89458400
H	2.75630100	-1.98476500	0.09933000
C	3.23333800	0.08770300	0.35964500
H	3.52971900	2.18060400	0.73931100

C	-1.39300500	0.91687600	1.56220800
C	-0.20606600	0.59279800	2.14209200
C	-1.83226900	-0.26544500	0.88149900
H	0.45031000	1.21851400	2.72608900
N	0.11821100	-0.71689300	1.88376600
C	-0.85553400	-1.26553400	1.10020700
C	-2.91725200	-0.61026900	0.07630700
H	1.00207300	-1.14708500	2.08724100
C	-0.94580800	-2.54254000	0.56331000
C	-3.02026000	-1.86554500	-0.47768600
H	-0.17727300	-3.28087200	0.75427100
C	-2.03461700	-2.82751500	-0.23005500
H	-3.87735200	-2.10355500	-1.09267700
H	-2.14307500	-3.81302200	-0.66381100
C	-2.03755600	2.25424900	1.61289100
H	-2.12468500	2.69330500	0.61513900
H	-3.04413500	2.21411600	2.03174800
H	-1.44574700	2.93544600	2.22655400
N	-0.15322200	-0.26331900	-2.03042700
H	-0.77296400	-1.01018700	-2.29146300
C	0.43912800	1.76975200	-1.27734800
C	0.42878700	3.21130400	-0.93146500
H	0.41295600	3.35374300	0.15463000
H	1.31528500	3.72651700	-1.30929100
H	-0.44883100	3.71236300	-1.34126800
Cl	-4.16999200	0.55974200	-0.26649700
Cl	4.93845200	-0.07331500	0.80373600

Entry: 4I-5I
E^{CP} (a.u.): -1396.27269

C	-1.06147700	-1.08008900	2.03956900
C	0.64896600	0.32981100	1.79316000
H	-2.06380500	-1.37669700	2.30761700
C	1.02151800	-0.92042900	1.26198100
C	1.50154200	1.42331100	1.76003400
C	2.26657500	-1.06510800	0.65149100
C	2.72730500	1.26951200	1.14701000
H	1.20921100	2.37684500	2.18211200
H	2.56022700	-2.01329400	0.21629000
C	3.09124600	0.03582700	0.59087300
H	3.41436800	2.10387500	1.09578900
C	-1.14941800	-0.58808900	-1.68112000
C	0.13054700	-0.31791300	-2.05592200
C	-1.60872800	0.57204600	-0.96961800
H	0.81981800	-0.94737200	-2.59675000
N	0.50354500	0.93300900	-1.63716900
C	-0.53802700	1.50226400	-0.96575400
C	-2.77895400	0.95559400	-0.30456100
H	1.42471000	1.32432300	-1.71617100
C	-0.61426600	2.74868500	-0.36201800
C	-2.86692300	2.19016700	0.30410100
H	0.22957300	3.42694200	-0.38476600
C	-1.78890400	3.08254200	0.27062900
H	-3.78099400	2.47804800	0.80686900
H	-1.89205100	4.04923200	0.74654600
C	-1.85849300	-1.86172700	-1.97060400
H	-2.12673500	-2.39309400	-1.05421300
H	-2.78304100	-1.70717300	-2.52980700
H	-1.21738200	-2.51885600	-2.56100500
N	-0.62164300	0.20222300	2.28389400
H	-1.21886400	0.97325900	2.52564200
C	-0.08445200	-1.80858400	1.43538200
C	-0.13872000	-3.22417200	0.99878700
H	0.07241100	-3.31169600	-0.07181800
H	0.59804900	-3.84072300	1.51992700
H	-1.12324200	-3.65769900	1.17867800
I	-4.44816300	-0.31929100	-0.12777600
I	4.92568100	-0.10286800	-0.43548000

Entry: 4Cl-6Cl
E^{CP} (a.u.): -1725.14553

C	-1.26923900	-0.75332600	-1.64388300
C	-0.01420100	-0.47543600	-2.08963800
C	-1.71363400	0.42565600	-0.95708400
H	0.66089600	-1.11450200	-2.63667500
N	0.35137900	0.80200400	-1.74633400
C	-0.66513200	1.37486300	-1.03877200
C	-2.85228200	0.80871400	-0.24548100
H	1.27538900	1.18536900	-1.83585700
C	-0.73761100	2.63747500	-0.46818400
C	-2.93769700	2.05160900	0.34014500
H	0.08448700	3.33588100	-0.56176600
C	-1.88175400	2.96304100	0.22361400
H	-3.83353800	2.32541700	0.88083600
H	-1.97927500	3.94040500	0.67816500
C	-1.97308500	-2.04690400	-1.84178100
H	-2.19229200	-2.53916700	-0.89109100
H	-2.92492000	-1.92583000	-2.36127100
H	-1.35337000	-2.72383100	-2.43234900
N	-0.41058000	0.24805300	2.18631100
H	-1.01332000	1.01322500	2.43255100
C	0.13871800	-1.78580500	1.40351200
C	0.09345100	-3.21798000	1.02261200
H	0.19174700	-3.33819600	-0.06145000
H	0.90419200	-3.78818200	1.48279900
H	-0.84887500	-3.67814000	1.32156100
Br	-4.32292800	-0.37612000	-0.03217100
Br	4.89565300	-0.06038400	-0.55408900

Entry: 4F-6F
E^{CP} (a.u.): -1004.42782

C	0.29604000	1.14248400	1.87295800
C	-1.38291500	-0.11013400	1.10310900
H	1.21288500	1.33941800	2.40669800
C	-1.53546300	1.19773500	0.59862900
C	-2.26096900	-1.13605800	0.77569700
C	-2.60088600	1.47995600	-0.25295600
C	-3.28918900	-0.80643100	-0.07335000
H	-2.16336300	-2.14324800	1.15822600
H	-2.73611300	2.47988600	-0.64834500
C	-3.47774200	0.47208400	-0.59453500
H	-4.32023100	0.64487900	-1.25018600
C	1.63319200	0.82896900	-1.47306700
C	0.52160400	0.57201000	-2.21292100
C	1.85250300	-0.34433900	-0.68378100
H	0.01501100	1.21178500	-2.91793400
N	0.04676100	-0.69200400	-1.94474500
C	0.83961500	-1.27402800	-0.99728300
C	2.76912700	-0.71001400	0.29451700
H	-0.82365300	-1.06681400	-2.27471900
C	0.74495600	-2.51849700	-0.38257500
C	2.69534000	-1.92364600	0.92938400
H	-0.04297200	-3.21305900	-0.64448200
C	1.68103300	-2.82599800	0.58009900
H	3.43235400	-2.17055600	1.68131300
H	1.64196500	-3.78686200	1.07652900
C	2.42179400	2.08595900	-1.44774900
H	2.44217300	2.51286200	-0.44083300
H	3.46009100	1.92528600	-1.74443900
H	1.99116800	2.82846400	-2.12083000
F	3.72670900	0.15485200	0.64177400
N	-0.27469500	-0.11442600	1.90063200
H	0.17790600	-0.94165100	2.24850900
C	-0.44914100	1.97966600	1.10650000
C	-0.19454900	3.40787800	0.80281500
H	-0.06058100	3.56142900	-0.27282200
H	-1.02722200	4.04421400	1.11361000
H	0.70462600	3.76791300	1.30382700
F	-4.15944400	-1.76090900	-0.42380500

Entry: 4Br-6Br
E^{CP} (a.u.): -5952.95772

C	-0.73639000	1.42909400	-1.80765300
C	1.15078400	0.38471600	-1.24350200
H	-1.70283700	1.53252300	-2.27554300
C	1.13588900	1.64647200	-0.61491100
C	2.17652300	-0.52772400	-1.04526900
C	2.18588800	1.99701700	0.23121500
C	3.17830200	-0.14891200	-0.18187200
H	2.18498600	-1.50056700	-1.51810200
H	2.19693400	2.96389400	0.72097100
C	3.20528300	1.09647600	0.45250000
H	4.03192100	1.33484200	1.10747400
C	-1.64024600	0.52892900	1.60400100
C	-0.45064700	0.54128900	2.26486300
C	-1.62721200	-0.65645800	0.79793000
H	-0.07177600	1.28029800	2.95326100
N	0.29640500	-0.56387000	1.93984800
C	-0.39506200	-1.31109900	1.03137600
C	-2.48100400	-1.24039200	-0.13828700
H	1.25862800	-0.71036400	2.18724000
C	-0.02394900	-2.48413400	0.38855800
C	-2.13041200	-2.39679600	-0.79577000
H	0.93216300	-2.95074000	0.59343700
C	-0.90361700	-3.01568800	-0.52728500
H	-2.81716100	-2.82751000	-1.51169900
H	-0.65278800	-3.93028100	-1.04856000
C	-2.68717800	1.57832000	1.70483700
H	-2.84901100	2.07755300	0.74589200
H	-3.65015200	1.17605700	2.02261600
H	-2.38684400	2.33918200	2.42735400
N	0.01086300	0.28404300	-1.99045000
H	-0.34584400	-0.57413300	-2.37380200
C	-0.08089600	2.29568500	-0.99132200
C	-0.53986000	3.62277900	-0.51656000
H	-0.65051500	3.62789300	0.57299600
H	0.16864400	4.41524900	-0.76993200
H	-1.50555500	3.88761600	-0.94802300
Cl	4.46601800	-1.28577500	0.15773400
Cl	-4.01359800	-0.49188200	-0.52293700

Entry: 4I-6I
E^{CP} (a.u.): -1396.27343

C	-1.17188000	1.65846900	-1.73844200
C	0.85676800	0.84042300	-1.30525600
H	-2.16885100	1.65257800	-2.15094800
C	0.72146200	2.07247600	-0.63451100
C	1.99294200	0.05322000	-1.18637300
C	1.76751100	2.52784000	0.16541600
C	2.99003400	0.52182700	-0.35864100
H	2.07400700	-0.90089400	-1.69122200
H	1.68910200	3.47652800	0.68428500
C	2.89678400	1.75071200	0.30836900
H	3.71498600	2.08308000	0.93354800
C	-1.52443200	0.57560500	1.82645500
C	-0.28571100	0.70954600	2.37348300
C	-1.46070300	-0.58283400	0.97940600
H	0.07001200	1.47094200	3.04971100
N	0.54491300	-0.29532700	1.94825900
C	-0.14361400	-1.09635300	1.08451200
C	-2.32819200	-1.24985200	0.10726200
H	1.54052300	-0.32568200	2.08243300
C	0.29654600	-2.21730500	0.39604700
C	-1.90434800	-2.36349300	-0.58712800
H	1.31450600	-2.57179000	0.50874100
C	-0.59883600	-2.84565900	-0.43841000
H	-2.58899500	-2.87419300	-1.25138000
H	-0.29888300	-3.72747600	-0.98959000
C	-2.66609400	1.48776700	2.09820800
H	-3.03101800	1.96867500	1.18757400
H	-3.51651100	0.96760100	2.54362700
H	-2.35715800	2.27321300	2.79045900

C	-1.00360400	1.61971200	-1.79349700
C	0.96760400	0.69805600	-1.30716500
H	-1.97927600	1.67763700	-2.24922400
C	0.86990100	1.92653600	-0.62283000
C	2.06165700	-0.14326000	-1.16567900
C	1.91065900	2.32205600	0.21510300
C	3.04909800	0.27158000	-0.30226300
H	2.12296100	-1.09528200	-1.67631100
H	1.86070200	3.26662500	0.74456200
C	2.99813300	1.49178900	0.37989500
H	3.81652800	1.76997700	1.03005300
C	-1.59437400	0.54519700	1.72963100
C	-0.38156000	0.66681900	2.33507600
C	-1.50518100	-0.62438200	0.90336800
H	-0.04587800	1.43172100	3.01766000
N	0.45341600	-0.35542800	1.95978500
C	-0.20450800	-1.15673200	1.07289100
C	-2.34341400	-1.29433300	0.00973900
H	1.43759400	-0.40636600	2.15479200
C	0.24675600	-2.29211700	0.41499700
C	-1.91511500	-2.41950200	-0.65679300
H	1.25459100	-2.65836500	0.57169600
C	-0.62238300	-2.91571500	-0.45064700
H	-2.58857300	-2.92441800	-1.33611300
H	-0.31193100	-3.80705100	-0.98002200
C	-2.73699200	1.47593600	1.92320800
H	-3.04714700	1.94457800	0.98630100
H	-3.61557100	0.97246400	2.33039300
H	-2.45697500	2.27116000	2.61655600
N	-0.17804700	0.54295200	-2.03511600
H	-0.45309800	-0.30154100	-2.50332600
C	-0.39935300	2.49615800	-0.94769300
C	-0.94674500	3.76503800	-0.41099900
H	-1.00931700	3.73167000	0.68162500
H	-0.31914700	4.62186500	-0.66868500
H	-1.94906600	3.95866800	-0.79407800
Br	4.51975100	-0.88978100	0.01987500
Br	-4.10110700	-0.66344800	-0.34435000

Entry: 4F-7F
E^{CP} (a.u.): -1004.42684

C	0.11190700	-0.86312300	2.07197000
C	1.59632000	0.29773100	0.89697600
H	-0.69822400	-1.00912200	2.76911800
C	1.72276400	-1.05540200	0.53399400
C	2.37903300	1.26338300	0.28778500
C	2.64303400	-1.42397100	-0.44981100
C	3.27296900	0.90420200	-0.68841400
H	2.75043600	-2.46321900	-0.73683600
C	3.40276800	-0.44537400	-1.05114100
H	3.88032200	1.67091800	-1.15049600
H	4.12405900	-0.71170300	-1.81261100
C	-1.59363600	-1.17468800	-1.23947800
C	-0.52019200	-1.02468200	-2.05979400
C	-1.81379500	0.11062200	-0.65033400
H	-0.02454300	-1.76581700	-2.66682200
N	-0.07394000	0.27774500	-2.03881100
C	-0.83957800	0.99281700	-1.15892100
C	-2.70674500	0.61869100	0.28551000
H	0.81077300	0.58470000	-2.40386800
C	-0.75488600	2.32406900	-0.76565100
C	-2.64699800	1.92548100	0.69680300
H	0.00227800	2.98172400	-1.17224100
C	-1.66644200	2.77296100	0.16432100
H	-3.36504800	2.28358400	1.42198400
H	-1.63272400	3.80432800	0.48981800
C	-2.35630000	-2.42172800	-0.98361800
H	-2.31894200	-2.69571800	0.07404000
H	-3.41137200	-2.31659000	-1.24416500
H	-1.94707400	-3.25255800	-1.56018500

N	-0.30261800	0.62085200	-1.99411000
H	-0.54874200	-0.24071000	-2.44704300
C	-0.58342600	2.57565200	-0.92441600
C	-1.16825200	3.82841500	-0.38923900
H	-1.17542400	3.81917100	0.70546400
H	-0.59747600	4.70840500	-0.69658200
H	-2.19634100	3.96370500	-0.72650700
I	4.67632600	-0.69257200	-0.02352800
I	-4.29404600	-0.58102400	-0.25707700

Entry: 4Cl-7Cl
E^{CP} (a.u.): -1725.14733

C	-0.14215000	0.67106100	-1.98836900
C	1.68339100	0.14380700	-0.83484900
H	-1.00024200	0.48333400	-2.61463400
C	1.42304000	1.51576200	-0.63841100
C	2.75948000	-0.46705200	-0.21054800
C	2.24590600	2.25998500	0.20488700
C	3.55863200	0.27090800	0.63299700
H	2.05589300	3.31546100	0.36125700
C	3.29679300	1.63102400	0.83834800
H	4.39892400	-0.20799400	1.11746500
H	3.94780000	2.19228200	1.49583800
C	-1.85715300	1.04104400	1.26583000
C	-0.79730300	1.29250400	2.08067500
C	-1.69477400	-0.31087800	0.81769300
H	-0.55040600	2.20476800	2.60071500
N	0.00099500	0.18057200	2.19592600
C	-0.51775700	-0.81570800	1.41758900
C	-2.37910700	-1.16672900	-0.04431100
H	0.91736400	0.16582400	2.60609900
C	-0.04807200	-2.10286200	1.19848600
C	-1.92376500	-2.44197000	-0.28842200
H	0.85563800	-2.45714500	1.67778200
C	-0.76181400	-2.90531300	0.33674600
H	-2.47909900	-3.08202400	-0.96023500
H	-0.42252400	-3.91335100	0.13687700
C	-2.93868600	2.00341800	0.93328700
H	-2.99280500	2.19811700	-0.14008500
H	-3.92072000	1.63387600	1.23416900
H	-2.76781600	2.95527900	1.43900800
N	0.73194000	-0.34733200	-1.67754400
H	0.57895100	-1.32484300	-1.85451200
C	0.25188600	1.83007300	-1.39613000
C	-0.39302200	3.16240100	-1.47551600
H	-0.67715300	3.52132600	-0.48133600
H	0.27765000	3.91106800	-1.90503300
H	-1.29460800	3.13260800	-2.08808000
Cl	3.08231900	-2.15478400	-0.51173700
Cl	-3.82646800	-0.62345900	-0.86202000

Entry: 4I-7I
E^{CP} (a.u.): -1396.27593

C	-0.18085900	0.61671800	-1.95409900
C	1.74033300	0.74038500	-0.83397700
H	-0.98139300	0.12504500	-2.48474700
C	1.16370000	2.02973400	-0.86978700
C	2.94262000	0.51385700	-0.17805300
C	1.80438600	3.08671500	-0.22833400
C	3.55739200	1.56850700	0.46526400
H	1.37324300	4.08087300	-0.25321600
C	2.98725000	2.84728900	0.43823800
H	4.49562300	1.40883200	0.98041300
H	3.50113600	3.65650300	0.94097500
C	-2.02835300	1.53887800	1.29149200
C	-1.00419100	2.17859900	1.91904600
C	-1.60696300	0.17489900	1.13155900
H	-0.93664600	3.21721300	2.20274900
N	0.02024500	1.30789600	2.18733200
C	-0.31382300	0.07493800	1.70459700

F	-3.63006900	-0.19523200	0.80746400
N	0.62445600	0.39532000	1.84912500
H	0.23592700	1.25752500	2.18562700
C	0.75868500	-1.78029700	1.30212900
C	0.50763800	-3.23872900	1.21619900
H	0.24788000	-3.52892400	0.19283400
H	1.38827000	-3.82108800	1.49897300
H	-0.31499400	-3.54104400	1.86491700
F	2.23452300	2.54187300	0.65104300

Entry: 4Br-7Br
E^{CP} (a.u.): -5952.96001

C	-0.18418800	0.55076700	-1.96897600
C	1.72266900	0.49462600	-0.82600700
H	-1.00486500	0.13930100	-2.53563900
C	1.22443400	1.81458100	-0.78500200
C	2.89711400	0.16106300	-0.16957700
C	1.91483700	2.78809100	-0.06611900
C	3.56641300	1.12796800	0.54747600
H	1.54167800	3.80497800	-0.02876600
C	3.07138200	2.43680600	0.59756900
H	4.48455400	0.87134200	1.05885800
H	3.62227600	3.18043900	1.15860900
C	-1.98059200	1.26836200	1.29158100
C	-0.94339600	1.79009200	2.00132700
C	-1.62430300	-0.09460700	1.01853500
H	-0.82719000	2.79323800	2.38073200
N	0.02474500	0.84134300	2.21538200
C	-0.36029400	-0.32316100	1.61393500
C	-2.19139200	-1.16115400	0.31950600
H	0.94137100	1.01959400	2.58448300
C	0.29843500	-1.54231900	1.55144300
C	-1.54907100	-2.37588100	0.23638100
H	1.26337000	-1.67967700	2.02362500
C	-0.31029400	-2.56334000	0.85747200
H	-2.01235600	-3.18757600	-0.30831900
H	0.17466100	-3.52837200	0.78597500
C	-3.21243800	2.00891600	0.91257400
H	-3.34315700	2.05915300	-0.17039300
H	-4.11265500	1.54167100	1.31582000
H	-3.16739500	3.03141800	1.29182900
N	0.86252100	-0.25249400	-1.57351000
H	0.87929800	-1.25607100	-1.62710700
C	0.00509200	1.82428600	-1.53177400
C	-0.86844500	3.00213800	-1.74882500
H	-1.17097800	3.44571800	-0.79511300
H	-0.36129800	3.78409600	-2.31995000
H	-1.77436400	2.72864400	-2.29111600
Br	3.55811900	-1.61222400	-0.27234200
Br	-3.84953600	-0.96244000	-0.58839400

Entry: 5F-5F
E^{CP} (a.u.): -1004.42492

C	-1.99113000	-0.89375100	-2.10139200
C	-0.69703700	0.86799700	-1.64582900
H	-2.34627100	-1.73780800	-2.67171000
C	-1.62107800	0.71012500	-0.59433700
C	0.25947200	1.87282700	-1.64279200
C	-1.57087800	1.57389600	0.50011300
C	0.30109700	2.71528200	-0.55283500
H	0.96804400	1.98115600	-2.45411900
H	-2.24758300	1.48276400	1.34073800
C	-0.59994700	2.54009000	0.49388700
H	1.03744900	3.50393700	-0.47807900
C	-0.21923100	-1.95208000	1.37448300
C	-0.45869700	-1.20222800	2.48556800
C	0.89710900	-1.34792800	0.71972100
H	-1.22021200	-1.33100100	3.23894100
N	0.45626100	-0.17970600	2.57788400
C	1.29739900	-0.24290300	1.49687200

C	-2.13194300	-0.98633400	0.55539700
H	0.93212700	1.56614200	2.51917300
C	0.41797500	-1.10221100	1.73488300
C	-1.41324000	-2.16362200	0.57429200
H	1.40433500	-1.13309600	2.18207200
C	-0.14668000	-2.21954500	1.16430100
H	-1.83234300	-3.05487000	0.12623800
H	0.39585700	-3.15650100	1.16415500
C	-3.30163300	2.19054200	0.88598400
H	-3.46045700	2.14531300	-0.19370700
H	-4.17189900	1.72406000	1.35148300
H	-3.29031900	3.24249400	1.17733900
N	0.91253000	-0.09838500	-1.51935100
H	1.00081200	-1.09921600	-1.53871400
C	-0.06153800	1.92379600	-1.59851100
C	-1.00492700	3.03262200	-1.87847400
H	-1.31868800	3.52443200	-0.95235300
H	-0.55302200	3.79984000	-2.51257700
H	-1.90173400	2.66973700	-2.38237100
I	3.80695000	-1.39880700	-0.18403700
I	-3.99002800	-1.00606000	-0.43758000

Entry: 5Cl-5Cl
E^{CP} (a.u.): -1725.14583

C	-0.36904100	2.57031800	-1.83001900
C	0.05562200	0.38324400	-1.97332700
H	-0.92184400	3.48126700	-1.99717600
C	1.08837900	0.99137200	-1.23149400
C	0.02919200	-0.98263300	-2.21436100
C	2.10761800	0.20383000	-0.69919600
C	1.04198100	-1.74939700	-1.68160000
H	-0.77606800	-1.44211000	-2.77399300
H	2.90510900	0.63387700	-0.10621500
C	2.05120800	-1.14927800	-0.92635900
H	1.05373700	-2.82169900	-1.82039000
C	-0.04205500	1.28423500	2.21483800
C	1.06986700	0.66380900	2.69573800
C	-0.82623900	0.26495700	1.59194600
H	1.90809700	1.08899600	3.22513600
N	1.01470000	-0.68647300	2.43485100
C	-0.13789400	-0.95517100	1.74747200
C	-2.02486600	0.29171800	0.88152800
H	1.76452500	-1.33790700	2.57738300
C	-0.63223300	-2.14777300	1.23988000
C	-2.48429100	-0.89351300	0.36130500
H	-0.09244700	-3.07884800	1.36107700
C	-1.82003400	-2.10794700	0.54287200
H	-2.23825600	-3.00784200	0.11299300
C	-0.40104000	2.71927900	2.31405400
H	-0.54744500	3.15713200	1.32215400
H	-1.33230600	2.86275300	2.86797700
H	0.37779300	3.29106500	2.82029200
N	-0.81934200	1.37177200	-2.33569300
H	-1.69133600	1.22852100	-2.80977400
C	0.80013200	2.38876100	-1.15712200
C	1.62699300	3.41296900	-0.47512500
H	1.78437000	3.15347900	0.57612300
H	2.61515800	3.50520900	-0.93314300
H	1.15258600	4.39452800	-0.50816300
H	-2.56641700	1.21527200	0.71869000
Cl	3.26143700	-2.18800500	-0.19194300
Cl	-3.94158700	-0.88572800	-0.61589600

Entry: 5I-5I
E^{CP} (a.u.): -1396.27582

C	-0.88904000	2.73540800	-1.99592400
C	-0.12973400	0.63655600	-1.93434200
H	-1.59688400	3.52250900	-2.20251800
C	0.85558500	1.48486000	-1.39066600
C	0.04938200	-0.73638400	-2.02164300

C	1.57306800	-1.63672700	-0.46601600
H	0.43164700	0.56567000	3.24807300
C	2.36147700	0.56707900	1.12969800
C	2.61300200	-0.81516800	-0.81508000
H	2.65373200	1.41962200	1.73026900
C	3.02415100	0.26795500	-0.04119200
H	3.85636900	0.86636500	-0.38613400
C	-0.95350000	-3.15348200	0.90942200
H	-1.40391400	-2.98070700	-0.07420900
H	-0.29022400	-4.01677800	0.81220100
H	-1.75310500	-3.42566900	1.59964700
N	-0.95268900	-0.11923500	-2.56297100
H	-0.39906300	-0.31110400	-3.37612900
C	-2.44182800	-0.41580300	-0.90823500
C	-3.54971300	-0.94017400	-0.07448100
H	-3.20046300	-1.18905800	0.93300500
H	-4.35077000	-0.20524700	0.04049600
H	-3.98512100	-1.84134000	-0.50818700
F	3.26972500	-1.04902600	-1.96127500
H	1.28841800	-2.46351700	-1.10518600
F	-0.48154200	3.34738900	1.56133500

Entry: 5Br-5Br
E^{CP} (a.u.): -5952.95922

C	-0.80650800	2.51039600	-2.00146100
C	-0.06705900	0.40499300	-1.92994400
H	-1.50161800	3.30490600	-2.22247900
C	0.90990700	1.24181500	-1.35425300
C	0.10089500	-0.96917600	-2.01917400
C	2.07038900	0.68080600	-0.82485400
C	1.25381200	-1.51086500	-1.49377600
H	-0.65784200	-1.60440600	-2.45916400
H	2.82727500	1.29702600	-0.35501600
C	2.20796800	-0.68459900	-0.89473900
H	1.41693200	-2.57966200	-1.52020500
C	-0.11221500	1.84327100	2.06622100
C	1.10243400	1.44743300	2.53544600
C	-0.77634800	0.65378700	1.63480200
H	1.90118500	2.04747600	2.94237300
N	1.22483300	0.07854000	2.45075000
C	0.08653800	-0.43101700	1.88876600
C	-2.00247000	0.42669700	1.01306600
H	2.06829800	-0.44181000	2.61050400
C	-0.25743800	-1.73492100	1.56278100
C	-2.31554700	-0.86683000	0.67062800
H	0.41640800	-2.56051800	1.75579200
C	-1.47375200	-1.94620900	0.95002500
H	-1.77446000	-2.94424700	0.66132100
C	-0.66554500	3.21708200	1.99760900
H	-0.91443000	3.48696100	0.96619200
H	-1.58348500	3.30983000	2.58355900
H	0.04482700	3.95396000	2.37459800
N	-1.10417900	1.20548900	-2.32482300
H	-1.97895600	0.88025900	-2.69228400
C	0.42031100	2.58300600	-1.41652000
C	1.13418100	3.79012900	-0.93554200
H	1.40968300	3.68946700	0.11885000
H	2.05962300	3.95983200	-1.49191200
H	0.51792500	4.68452400	-1.03448100
H	-2.67523800	1.24419300	0.78547200
Br	3.72655500	-1.50182100	-0.08522900
Br	-3.93449800	-1.20248700	-0.27474600

Entry: 5F-6F
E^{CP} (a.u.): -1004.42701

C	-0.00846200	1.05805500	2.01447600
C	-1.37854000	-0.44226000	1.08976800
H	0.85068300	1.39051200	2.57520300
C	-1.83390600	0.84832900	0.74870100
C	-2.01101200	-1.59298300	0.63430800

C	2.03605900	0.93730000	-0.89205900
C	1.22219200	-1.26412500	-1.52645800
H	-0.71215300	-1.38219100	-2.44081700
H	2.79341700	1.57121500	-0.44602100
C	2.18972900	-0.42893400	-0.95638300
H	1.38615500	-2.33307200	-1.55655300
C	-0.05064000	2.11744500	2.05001700
C	1.19302200	1.76311600	2.47356000
C	-0.69891100	0.90251500	1.66669900
H	1.99119000	2.39162000	2.83645500
N	1.34942200	0.39637000	2.40833700
C	0.20390100	-0.15337400	1.90246200
C	-1.94382000	0.63060100	1.10260000
H	2.21569700	-0.09701600	2.53275300
C	-0.11781700	-1.47204800	1.61567200
C	-2.24296600	-0.67837800	0.79884800
H	0.58628900	-2.27548700	1.79431100
C	-1.35428000	-1.72698100	1.06184000
H	-1.63343100	-2.74154400	0.81079300
C	-0.64360900	3.47473500	1.98360300
H	-0.93653400	3.72422900	0.95865700
H	-1.54209700	3.55043600	2.60124700
H	0.05928300	4.23526100	2.32608900
N	-1.18461100	1.42554400	-2.30260000
H	-2.05635600	1.09483300	-2.67210800
C	0.35268000	2.82173100	-1.44575800
C	1.06928000	4.03796000	-0.99233900
H	1.37383400	3.94895800	0.05514100
H	1.97808400	4.20985500	-1.57477700
H	0.44360200	4.92671300	-1.08238400
H	-2.63962000	1.43344600	0.88891800
I	-4.06637000	-1.11991000	-0.16037100
I	3.88768600	-1.31452700	-0.07675600

Entry: 5Cl-6Cl
***E*^CP^ (a.u.):** -1725.14485

C	-0.30165300	1.54368300	-1.96048400
C	1.11880000	-0.02757600	-1.26437400
H	-1.20512500	1.92591800	-2.40901200
C	1.64579500	1.23314600	-0.91795800
C	1.75193400	-1.21300600	-0.92040400
C	2.84828700	1.29841800	-0.21722300
C	2.92357000	-1.10092500	-0.20818400
H	1.34087100	-2.18149800	-1.17191900
H	3.27415400	2.25773700	0.05342400
C	3.48552400	0.13053300	0.14200400
H	4.41809200	0.14587600	0.68923200
C	-1.16701300	1.65665300	1.62590000
C	0.03724500	1.38207000	2.19704800
C	-1.64216600	0.42027300	1.09041900
H	0.72189700	2.05206200	2.69299300
N	0.33466200	0.04512400	2.06837000
C	-0.67549300	-0.56462400	1.37676700
C	-2.77815600	0.07490600	0.35893200
H	1.22070400	-0.37495000	2.28472700
C	-0.82015200	-1.88014500	0.96152700
C	-2.89972500	-1.22850400	-0.06000400
H	-0.07006900	-2.62781400	1.19028000
C	-1.94352400	-2.20708300	0.23191700
H	-2.10341200	-3.21909900	-0.11432400
C	-1.85457700	2.96686600	1.53364500
H	-2.00850000	3.25732500	0.48957100
H	-2.83948700	2.94288300	2.00664200
H	-1.27479400	3.75522100	2.01448300
N	-0.06049800	0.18714800	-1.91874900
H	-0.72772600	-0.52641300	-2.15212000
C	0.71889600	2.22203500	-1.37218400
C	0.85260000	3.68564500	-1.18018900
H	0.90819400	3.93294800	-0.11536400
H	1.76047100	4.07657400	-1.64636700
H	0.00372100	4.22357900	-1.60291400

C	-2.95069800	0.98205700	-0.07222100
C	-3.10255800	-1.40507700	-0.17902600
H	-1.68436900	-2.59105600	0.89432000
H	-3.31680500	1.96535100	-0.34358400
C	-3.58405300	-0.14903700	-0.54341500
H	-4.45615700	-0.09354700	-1.18044600
C	1.36800500	1.49159000	-1.41450700
C	0.22526300	1.11567300	-2.05115000
C	1.89651600	0.30796700	-0.81536800
H	-0.47684200	1.72292800	-2.60098800
N	0.01894100	-0.23587200	-1.91002400
C	1.02216000	-0.75229200	-1.13114800
C	3.01424100	0.06373900	-0.01605600
H	-0.83567400	-0.71308100	-2.13533700
C	1.23905100	-2.04348100	-0.67377000
C	3.20140900	-1.21729300	0.43623100
H	0.56578000	-2.85142200	-0.93181400
C	2.34144300	-2.26995300	0.12566700
H	2.56905300	-3.25601900	0.50747300
C	1.95478300	2.85049600	-1.32466500
H	2.05367900	3.17142600	-0.28314000
H	2.95416000	2.89197400	-1.76551400
H	1.33519400	3.58551300	-1.83937100
N	-0.27875000	-0.28989900	1.88319400
H	0.34349200	-1.03035000	2.15285700
C	-0.93871200	1.78955500	1.34905000
C	-1.01413600	3.26314300	1.21037500
H	-0.97148200	3.55610100	0.15651700
H	-1.94723100	3.66407500	1.61472100
H	-0.18885500	3.75554800	1.72550800
F	4.26049700	-1.48658700	1.21029700
H	3.71743600	0.84273300	0.25060600
F	-3.74251600	-2.48226400	-0.64726400

Entry: 5Br-6Br
***E*^CP^ (a.u.):** -5952.95752

C	-0.50089200	1.92905400	-1.95278900
C	1.02694200	0.42224300	-1.35087500
H	-1.44846400	2.27213700	-2.33735700
C	1.52828300	1.70599200	-1.05376100
C	1.72591600	-0.73507900	-1.03991800
C	2.76940000	1.82370000	-0.43136900
C	2.93128600	-0.57304500	-0.39677300
H	1.33198700	-1.71891400	-1.25781000
H	3.17675000	2.80116400	-0.19944900
C	3.46770800	0.68355400	-0.09533700
H	4.42931100	0.74538200	0.39586000
C	-1.04516100	2.07405700	1.70441200
C	0.22439400	1.90329300	2.16306400
C	-1.48161200	0.78754600	1.26140400
H	0.90840000	2.63706700	2.56009100
N	0.59889000	0.58331800	2.05812700
C	-0.42716300	-0.11885700	1.48918300
C	-2.65598100	0.33896600	0.65697500
H	1.53610100	0.23894900	2.17102000
C	-0.52170900	-1.45871800	1.14257000
C	-2.72720200	-0.98639900	0.29737700
H	0.29559900	-2.14608800	1.32642900
C	-1.68290500	-1.88679000	0.53466800
H	-1.79926000	-2.92182700	0.24403600
C	-1.83147100	3.32893000	1.63828500
H	-2.09866400	3.57145500	0.60494900
H	-2.76661600	3.25138300	2.19852700
H	-1.27162900	4.17237000	2.04313500
N	-0.20399800	0.58405900	-1.92002700
H	-0.85700600	-0.15789500	-2.09836600
C	0.53342500	2.65349600	-1.44873300
C	0.62109500	4.12357700	-1.27964900
H	0.75452100	4.38569100	-0.22532300
H	1.46971100	4.54620500	-1.82322200
H	-0.28254400	4.62082200	-1.63317600

H	-3.54046500	0.80500600	0.11704000
Cl	-4.29991100	-1.69600100	-1.00335700
Cl	3.73527500	-2.56431900	0.30472900

Entry: 5I-6I
E^{CP} **(a.u.):** -1396.27359

C	-0.70255300	2.11886900	-1.94909900
C	0.93718100	0.71682300	-1.38780500
H	-1.68597100	2.39682300	-2.29449000
C	1.37813700	2.03482600	-1.15270200
C	1.72030400	-0.38843900	-1.08583100
C	2.64240200	2.24185800	-0.60532200
C	2.95145700	-0.14299200	-0.51751300
H	1.36702800	-1.39690100	-1.25887400
H	3.00312400	3.24787000	-0.42286800
C	3.42531800	1.15468000	-0.28148500
H	4.40561600	1.29560700	0.15419900
C	-0.95113300	2.38219500	1.77440000
C	0.34432700	2.25168700	2.16919900
C	-1.37639200	1.07660700	1.37816800
H	1.02808200	3.00872400	2.52013300
N	0.74650600	0.93935500	2.06700900
C	-0.28979800	0.20033300	1.56891100
C	-2.56589100	0.58950700	0.83797700
H	1.69345400	0.61542500	2.15470500
C	-0.37107800	-1.14901200	1.25731500
C	-2.62810500	-0.74491600	0.50328900
H	0.46948700	-1.81444500	1.41583000
C	-1.54988500	-1.61552500	0.71402900
H	-1.64524600	-2.66077600	0.45107800
C	-1.78032800	3.61087500	1.74321200
H	-2.14336800	3.81746600	0.73175800
H	-2.66143700	3.51856200	2.38321000
H	-1.21732100	4.48182700	2.07953500
N	-0.32966400	0.79435800	-1.89340200
H	-0.94349400	0.01365000	-2.04228800
C	0.31386600	2.91405100	-1.51973600
C	0.33476000	4.39222400	-1.41007600
H	0.52216700	4.70505200	-0.37809900
H	1.12379100	4.83298500	-2.02450300
H	-0.61364000	4.82967000	-1.72295500
H	-3.40893500	1.25028000	0.67403400
I	-4.38008300	-1.50525400	-0.38587800
I	4.13089900	-1.78040100	0.08668500

Entry: 5Cl-7Cl
E^{CP} **(a.u.):** -1725.14689

C	-0.38233900	0.61559300	2.16537900
C	-1.59437300	-0.65533000	0.80425100
H	0.41056600	0.83499800	2.86256000
C	-2.10570400	0.65709400	0.74566800
C	-2.14804100	-1.66718200	0.03621200
C	-3.17285100	0.94093800	-0.10507500
C	-3.19330600	-1.37707400	-0.81000700
H	-3.57644400	1.94535000	-0.15960400
C	-3.70055900	-0.07257000	-0.87688300
H	-3.62545300	-2.16795900	-1.40789000
H	-4.53207900	0.12771900	-1.53986300
C	0.95929800	2.37485500	-0.93846900
C	-0.16633000	2.19126100	-1.67974300
C	1.54023400	1.07993800	-0.77224200
H	-0.89428800	2.91977400	-2.00193800
N	-0.31191700	0.86394300	-2.01260800
C	0.71205700	0.15608000	-1.44140200
C	2.67072900	0.62675400	-0.09427100
H	-1.15431300	0.45684900	-2.37991500
C	0.98818300	-1.20283200	-1.44955700
C	2.92338400	-0.72478800	-0.10150300
H	0.34677200	-1.90754900	-1.96421500
C	2.10331600	-1.63989800	-0.76650000

H	-3.48171300	1.01313500	0.46513700
Br	3.88213700	-2.12405700	0.15349800
Br	-4.29318900	-1.63525100	-0.56588700

Entry: 5F-7F
E^{CP} **(a.u.):** -1004.42638

C	-0.32331800	0.61057500	2.15393600
C	-1.47830300	-0.76366200	0.84745600
H	0.44106400	0.88863100	2.86206400
C	-1.98285500	0.53587300	0.65894200
C	-1.99325300	-1.82568500	0.12444000
C	-3.01123800	0.75260100	-0.26040300
C	-2.99464500	-1.61263800	-0.78843400
H	-3.40779000	1.74959500	-0.41147400
C	-3.50366100	-0.31786300	-0.97358800
H	-3.38833500	-2.45558000	-1.34028500
H	-4.30339600	-0.17086900	-1.68756700
C	1.14191000	1.89811900	-1.09146000
C	0.06837200	1.56823900	-1.86009000
C	1.74373800	0.66344900	-0.69936100
H	-0.65688400	2.21676200	-2.32671200
N	-0.02247900	0.20411400	-2.00112700
C	0.98158800	-0.37744400	-1.26776500
C	2.84374800	0.35536200	0.10243400
H	-0.83839200	-0.27799600	-2.33690200
C	1.28668700	-1.71268600	-1.04931300
C	3.12016300	-0.97125700	0.31277400
H	0.69833100	-2.50664000	-1.49143800
C	2.36727500	-2.00482700	-0.24183900
H	2.65738800	-3.02654800	-0.03780100
C	1.60583000	3.25319600	-0.70803100
H	1.68580800	3.35269900	0.37896700
H	2.59454000	3.47658800	-1.11765800
H	0.91946400	4.02369000	-1.06176400
N	-0.47987600	-0.70379400	1.77494000
H	0.13741500	-1.46117400	2.00457000
C	-1.22373800	1.39998100	1.50778700
C	-1.37491000	2.87198100	1.59630000
H	-1.16579900	3.33925900	0.62815700
H	-2.38932200	3.16060900	1.88208000
H	-0.68699500	3.30084400	2.32546600
F	4.16378200	-1.30456800	1.08359000
H	3.46453100	1.11979900	0.55249900
F	-1.48439100	-3.04785400	0.31400900

Entry: 5Br-7Br
E^{CP} **(a.u.):** -5952.95982

C	-0.46487700	0.91233500	2.16115000
C	-1.80470900	-0.25099300	0.82015300
H	0.38006300	1.07085300	2.81207600
C	-2.27931400	1.07727800	0.87188500
C	-2.44552700	-1.20306700	0.04229100
C	-3.39564400	1.43799400	0.12037500
C	-3.54155700	-0.83393900	-0.70554800
H	-3.76922600	2.45491100	0.15293700
C	-4.01033300	0.48560800	-0.66444200
H	-4.04665700	-1.57378900	-1.31196100
H	-4.88127200	0.74615300	-1.25176000
C	0.64721600	2.84538800	-0.97743300
C	-0.52452400	2.69668600	-1.65192200
C	1.22662800	1.54123600	-0.89734900
H	-1.26549300	3.44120900	-1.89874600
N	-0.70157700	1.38364200	-2.02473200
C	0.35081100	0.64865900	-1.54841000
C	2.39115400	1.05408500	-0.30581300
H	-1.56612800	0.99640000	-2.36039900
C	0.61514100	-0.71032900	-1.62758200
C	2.63056500	-0.29871400	-0.37658500
H	-0.06174100	-1.39107400	-2.12951200
C	1.76458600	-1.18101100	-1.02853500

H	2.35555800	-2.69109400	-0.74055300
C	1.48800800	3.64206800	-0.37934300
H	1.60226800	3.57419800	0.70722400
H	2.47254200	3.88715400	-0.78587800
H	0.82300100	4.47996800	-0.59186000
N	-0.55425700	-0.66214300	1.68197700
H	0.04175500	-1.45140600	1.85573800
C	-1.31209000	1.45231900	1.62980200
C	-1.46704200	2.90949700	1.85545400
H	-1.27643200	3.46948800	0.93342300
H	-2.47830300	3.16231200	2.18325000
H	-0.77077800	3.27223800	2.61199300
H	3.33142200	1.30829500	0.42679400
Cl	-1.49436600	-3.27947800	0.15376900
Cl	4.32566000	-1.33659700	0.75061600

Entry: 5I-7I
E^{CP} (a.u.): -1396.27599

C	-0.43686700	1.19718700	2.14332600
C	-1.96610700	0.20754900	0.86151400
H	0.45980300	1.25242200	2.73984000
C	-2.30514100	1.57375400	0.98269900
C	-2.74192400	-0.65560000	0.09963900
C	-3.42411300	2.06631100	0.31573400
C	-3.84149900	-0.15005000	-0.56284300
H	-3.69354300	3.11271300	0.40215700
C	-4.17645100	1.20562500	-0.45476900
H	-4.45827300	-0.81013200	-1.15853700
H	-5.05186200	1.56837100	-0.97789900
C	0.61564400	3.19165400	-1.02322300
C	-0.58685000	3.13421200	-1.65666800
C	1.10528800	1.84924800	-0.98056100
H	-1.28300100	3.93096000	-1.86762700
N	-0.86747600	1.84158000	-2.03639600
C	0.14846600	1.02945000	-1.61187500
C	2.25292200	1.27328400	-0.43782000
H	-1.75592900	1.52144400	-2.37861800
C	0.31840300	-0.34202900	-1.72414700
C	2.40288000	-0.09304900	-0.53578600
H	-0.41771900	-0.96578300	-2.21694900
C	1.45461700	-0.89971900	-1.17604700
H	1.61035100	-1.96884200	-1.23725300
C	1.29936500	4.38370900	-0.46675400
H	1.54918400	4.24054200	0.58895700
H	2.23849600	4.59255100	-0.98592300
H	0.67415900	5.27408100	-0.54245700
N	-0.82935600	0.00209900	1.58425000
H	-0.32177700	-0.86424000	1.62500800
C	-1.31346500	2.18292100	1.81201800
C	-1.27158400	3.61440700	2.19576200
H	-1.20051800	4.25595300	1.31192100
H	-2.17350200	3.91260300	2.73637100
H	-0.41499100	3.83170000	2.83413600
H	2.99952500	1.88499500	0.05433900
I	4.09319000	-1.01189000	0.32125700
I	-2.24572000	-2.69166200	-0.02774000

Entry: 6Cl-6Cl
E^{CP} (a.u.): -1725.14579

C	0.28262800	2.55328800	-1.29709600
C	0.97558400	0.43622300	-1.22244300
H	-0.40613400	3.35743900	-1.50158600
C	1.98936300	1.25349900	-0.68191100
C	1.09177000	-0.94429700	-1.28431000
C	3.15722800	0.66268700	-0.20440300
C	2.24946000	-1.48330700	-0.77541600
H	0.31199400	-1.57282600	-1.69386300
H	3.95382600	1.27168000	0.20676700
C	3.28354700	-0.70906100	-0.24502100
H	4.17050300	-1.20114800	0.12939100

H	2.00195200	-2.23585900	-1.05881200
C	1.21997400	4.08941800	-0.40957400
H	1.41753400	3.98108400	0.66134100
H	2.17122700	4.35114500	-0.88008200
H	0.54250000	4.93432700	-0.53831100
N	-0.70495100	-0.32987600	1.61875200
H	-0.13169300	-1.14669300	1.73099400
C	-1.40554600	1.80067600	1.74112700
C	-1.51171500	3.24159000	2.07347100
H	-1.43740300	3.85742600	1.17183900
H	-2.46796500	3.47769400	2.54702600
H	-0.71824100	3.55245100	2.75350300
H	3.08285500	1.71562900	0.20098600
Br	4.19072100	-1.00983600	0.44660800
Br	-1.79582400	-2.98232500	0.02245500

Entry: 6F-6F
E^{CP} (a.u.): -1004.42701

C	-0.88297700	-2.42028500	-1.02770400
C	-1.00951600	-0.20063300	-1.20644900
H	-0.43747700	-3.39444000	-1.15259600
C	-2.15698700	-0.65599400	-0.52609500
C	-0.77144300	1.14710600	-1.43612500
C	-3.09406100	0.26979300	-0.07283000
C	-1.71036100	2.01685000	-0.94367600
H	0.11186600	1.51173100	-1.94369400
H	-3.98519000	-0.06104500	0.44757900
C	-2.86501200	1.61379800	-0.27761500
H	-3.55594700	2.37050800	0.06831000
C	0.22422200	0.31112000	2.01513100
C	0.15288700	1.65555600	1.84208600
C	1.31585800	-0.13931600	1.20579200
H	-0.54321500	2.36239500	2.26429100
N	1.14412300	2.07801200	0.97741100
C	1.85415400	0.99149400	0.55528500
C	1.86615400	-1.39363500	0.95735400
H	1.23140700	3.00885400	0.61434200
C	2.90823500	0.89404900	-0.34578000
C	2.91676300	-1.50914200	0.07050000
H	3.33632800	1.74690100	-0.85534800
C	3.40990900	-0.36792100	-0.55941100
C	-0.66081100	-0.54901800	2.83699400
H	-1.11587300	-1.33118900	2.22105300
H	-0.11377400	-1.04796600	3.64118600
H	-1.46704000	0.03004100	3.28792300
C	-2.05395800	-2.08148400	-0.42711700
C	-3.04860300	-2.98174900	0.20546700
H	-3.23371800	-2.70200800	1.24693400
H	-4.01352600	-2.93939100	-0.30653400
H	-2.71242300	-4.01850700	0.19459300
N	-0.26129800	-1.29577500	-1.53276100
H	0.70115900	-1.25965700	-1.82074400
H	1.46625400	-2.27238400	1.44958500
F	4.42763400	-0.51294400	-1.41417000
H	3.38160000	-2.46178500	-0.14476900
F	-1.49395900	3.33280600	-1.08799700

Entry: 6Br-6Br
E^{CP} (a.u.): -5952.95850

C	0.12401600	2.83463800	-1.46626800
C	1.01139000	0.80791400	-1.20931700
H	-0.65226800	3.54967500	-1.68810600
C	1.99555300	1.76511100	-0.88896600
C	1.24150600	-0.55535000	-1.09987900
C	3.25024200	1.33433200	-0.46274300
C	2.48431100	-0.93245200	-0.64835300
H	0.47555000	-1.28505600	-1.32895800
H	4.02502500	2.05184100	-0.21916900
C	3.49177000	-0.01676000	-0.33535000
H	4.45120600	-0.37923400	0.00793000

C	0.16077400	0.08878000	2.16390800
C	0.52126400	-1.21873500	2.08174000
C	-1.06848600	0.20950300	1.44355700
H	1.39770600	-1.71062000	2.47312800
N	-0.41670100	-1.92894500	1.36327300
C	-1.38908300	-1.06865500	0.93812700
C	-1.90930500	1.28239700	1.15839100
H	-0.31243400	-2.88373900	1.07281700
C	-2.50188400	-1.28978300	0.13780700
C	-3.02151800	1.08005900	0.36910900
H	-2.74956300	-2.26482900	-0.25984700
C	-3.29473700	-0.19536100	-0.13143400
C	0.90010900	1.19563500	2.81698800
H	1.10237400	1.99617800	2.09814900
H	0.33615100	1.63620200	3.64295800
H	1.85830600	0.85219300	3.20778700
C	1.52441200	2.60507600	-0.74439800
C	2.27627500	3.80442400	-0.30059400
H	2.58028600	3.71965500	0.74674000
H	3.19078700	3.94402300	-0.88278100
H	1.67829400	4.71008200	-0.40086700
N	-0.04547200	1.25413100	-1.61702800
H	-0.96532300	0.94073600	-1.87266800
H	-1.68415700	2.27084400	1.54164100
H	-3.69534900	1.89183700	0.13211400
Cl	-4.70730100	-0.40814200	-1.13995400
Cl	2.41927300	-3.22763800	-0.75411700

Entry: 6I-6I
E^{CP} **(a.u.):** -1396.27448

C	-0.08122300	3.03685800	-1.55823600
C	1.03346800	1.13713200	-1.22288400
H	-0.93667800	3.64846700	-1.79765300
C	1.90994400	2.21371800	-0.97978300
C	1.41961200	-0.18435800	-1.05392700
C	3.21536400	1.94912900	-0.57195300
C	2.71113100	-0.40210800	-0.62618500
H	0.73116600	-1.00125700	-1.23067800
H	3.91025000	2.76023900	-0.38728100
C	3.61243400	0.64171900	-0.38926000
H	4.61752000	0.41662700	-0.05899200
C	0.54589600	1.38104000	2.24811100
C	1.19582800	0.19626500	2.39424300
C	-0.71931100	1.07016900	1.66116900
H	2.18596800	0.00589500	2.77782400
N	0.40907600	-0.84105100	1.94375200
C	-0.76300200	-0.32658000	1.46394800
C	-1.80254200	1.84675800	1.25877400
H	0.72349400	-1.78869800	1.83148200
C	-1.84144300	-0.95346000	0.85351500
C	-2.88282200	1.23990200	0.65289900
H	-1.86394600	-2.02487000	0.69933700
C	-2.88722400	-0.14622600	0.45145200
C	1.04186500	2.73722700	2.58397400
H	1.02148600	3.38498700	1.70148800
H	0.42984700	3.21817100	3.35128700
H	2.06909100	2.70321000	2.94767100
C	1.17550600	3.42007600	-1.20398900
C	1.70901200	4.79893200	-1.08137000
H	2.15030000	4.96960000	-0.09512300
H	2.49512500	4.99309300	-1.81563900
H	0.92717200	5.54396600	-1.22868800
N	-0.16916200	1.66345900	-1.60571800
H	-1.03136300	1.14781500	-1.64615100
H	-1.79058200	2.92049600	1.40771200
H	-3.73326600	1.82901100	0.33544600
I	3.32165800	-2.37861000	-0.23301400
I	-4.55071400	-1.02555600	-0.49229300

Entry: 6Cl-7Cl
E^{CP} **(a.u.):** -1725.14811

C	0.45212900	0.85374400	2.25957600
C	0.99288100	-0.39085300	2.33733000
C	-0.81003300	0.69851500	1.60697000
H	1.94674100	-0.69682100	2.73729500
N	0.13853000	-1.32007900	1.78377800
C	-0.96219900	-0.67073000	1.30105100
C	-1.81150700	1.59219800	1.23654100
H	0.38187000	-2.27650600	1.59661400
C	-2.06425000	-1.15503500	0.60908100
C	-2.91717200	1.12731300	0.55509900
H	-2.17777900	-2.20328200	0.36608500
C	-3.02135900	-0.23213600	0.24509400
C	1.04528100	2.13345200	2.71646000
H	1.16394700	2.82546900	1.87659100
H	0.41600900	2.63175100	3.45829600
H	2.02816500	1.97821300	3.16195500
C	1.40512800	3.05614100	-1.06563000
C	2.07902900	4.36089000	-0.85539500
H	2.49121300	4.44027000	0.15469500
H	2.91362000	4.49928000	-1.54744900
H	1.38883400	5.19218400	-0.99863300
N	-0.11389600	1.48362500	-1.59047700
H	-1.02673700	1.06976600	-1.67171100
H	-1.71945400	2.64628800	1.47165200
H	-3.71093800	1.80036400	0.26026200
Br	2.82199200	-2.78565200	-0.37754500
Br	-4.55021400	-0.83305100	-0.70823400

Entry: 6F-7F
E^{CP} **(a.u.):** -1004.42616

C	0.52513300	0.80209800	2.12642500
C	2.01416400	0.24125100	0.57863600
H	-0.02519700	1.46900200	2.77045300
C	1.33414400	-0.92802300	0.96511100
C	2.95884600	0.20228800	-0.43193200
C	1.65288900	-2.14383000	0.35652800
C	3.25904100	-0.98782900	-1.04426300
H	1.14430600	-3.05292900	0.65390700
C	2.60860800	-2.16227800	-0.63559200
H	4.00577700	-1.00134100	-1.82656800
H	2.87170700	-3.09574900	-1.11576600
C	-2.44305700	-1.50131900	-0.67163500
C	-1.40114600	-1.96792400	-1.40886800
C	-2.21625000	-0.09745200	-0.50251900
H	-1.19585600	-2.97369800	-1.73926600
N	-0.55169700	-0.93345100	-1.74555400
C	-1.01567500	0.21599600	-1.17210700
C	-2.90247900	0.90713200	0.17506500
H	0.38150300	-1.05716700	-2.09875200
C	-0.48141500	1.49736600	-1.17120900
C	-2.37981100	2.18312900	0.19650400
H	0.44683200	1.75331500	-1.66634800
C	-1.18367700	2.44294800	-0.46702000
C	-3.59622900	-2.26561000	-0.13636500
H	-3.67343400	-2.16034800	0.95008700
H	-4.54305700	-1.91381500	-0.55431400
H	-3.50882500	-3.32861500	-0.36150100
N	1.53088200	1.28182800	1.31725000
H	1.74269700	2.24977500	1.15775300
C	0.37618600	-0.53924100	1.95335600
C	-0.61131300	-1.43323900	2.60572400
H	-1.22806000	-1.93548900	1.85313100
H	-0.12864700	-2.21176400	3.20192400
H	-1.27761900	-0.87287600	3.26223200
H	-3.83109200	0.68735200	0.68846900
H	-2.87101200	2.99360300	0.71744600
F	-0.67861200	3.68276000	-0.39219700
F	3.55060200	1.34161100	-0.80666900

Entry: 6Br-7Br
E^{CP} **(a.u.):** -5952.96154

C	0.42713300	0.57296400	2.17386900
C	1.73878400	-0.47859500	0.72241700
H	0.07475900	1.41062100	2.75461400
C	0.73373000	-1.37884800	1.13284400
C	2.66599100	-0.84184200	-0.24009100
C	0.70349600	-2.66432300	0.59564500
C	2.62202100	-2.10936900	-0.77659100
H	-0.05916900	-3.36811900	0.90721900
C	1.64622300	-3.01778700	-0.34725700
H	3.35239100	-2.39424600	-1.52173800
H	1.64174800	-4.01376000	-0.77077800
C	-2.95450700	-1.12250200	-0.83206200
C	-2.02442700	-1.83174000	-1.52677000
C	-2.38216800	0.16868300	-0.60462300
H	-2.06791500	-2.85057200	-1.87758100
N	-0.91801700	-1.05105600	-1.78141300
C	-1.10358800	0.17069900	-1.19796700
C	-2.82064600	1.30535000	0.07116600
H	-0.03133300	-1.40197800	-2.09949700
C	-0.25385000	1.26444100	-1.12351700
C	-1.98241000	2.39464900	0.17102600
H	0.73435600	1.26299700	-1.56571900
C	-0.71709700	2.34923600	-0.41754500
C	-4.29916700	-1.56900800	-0.39215400
H	-4.41960000	-1.46653700	0.69026700
H	-5.09191100	-0.97525800	-0.85450800
H	-4.47548300	-2.61364800	-0.64846300
N	1.55252600	0.69330000	1.39005000
H	2.03922100	1.55061600	1.19405100
C	-0.09890200	-0.67621200	2.05911600
C	-1.31459900	-1.21423000	2.71593600
H	-2.02051300	-1.58948800	1.96879700
H	-1.08380500	-2.04541300	3.38713600
H	-1.82279800	-0.44489900	3.29773500
H	-3.80335500	1.32940900	0.52731800
H	-2.28390600	3.28648500	0.70279900
Cl	0.35297800	3.72093700	-0.20247800
Cl	3.84464900	0.33192700	-0.76533100

Entry: 6I-7I
E^{CP} (a.u.): -1396.27826

C	-0.59954600	-0.05989300	2.30566100
C	0.52377400	-1.44630000	0.97583600
H	-0.76461700	0.84608300	2.86716500
C	-0.73672500	-2.02229400	1.25215400
C	1.41640000	-2.06157200	0.10970400
C	-1.06784600	-3.25082600	0.68676300
C	1.06746900	-3.27230900	-0.45520100
H	-2.02863800	-3.70598500	0.89704600
C	-0.16498600	-3.86568600	-0.15548200
H	1.75540500	-3.76876500	-1.12710100
H	-0.40280400	-4.82360600	-0.60000200
C	-3.89652200	-0.74421000	-1.09077900
C	-3.12023200	-1.69245400	-1.68252700
C	-3.02602400	0.34892100	-0.78756300
H	-3.39774900	-2.66964400	-2.04485000
N	-1.82347600	-1.24537900	-1.80163300
C	-1.73623400	-0.00618800	-1.23045600
C	-3.21472000	1.57777300	-0.15915600
H	-1.03475000	-1.82982100	-2.01963500
C	-0.63640400	0.82028600	-1.05086500
C	-2.13021500	2.40504200	0.04096700
H	0.35249300	0.52648100	-1.38440500
C	-0.86047800	2.01358900	-0.39906200
C	-5.35167500	-0.80367200	-0.80774900
H	-5.55716300	-0.65493700	0.25643100
H	-5.89923200	-0.02544600	-1.34558600
H	-5.77458000	-1.76599400	-1.09605900
N	0.59476100	-0.26632500	1.65405800
H	1.32632500	0.41770200	1.55097000

C	-0.12442500	0.12946600	2.25038500
C	1.11447900	-1.10120100	0.87552400
H	-0.38183400	1.00521400	2.82486100
C	-0.05453000	-1.83017300	1.18334000
C	2.04591400	-1.59915500	-0.02154500
C	-0.24977800	-3.08563000	0.61208200
C	1.83617100	-2.83511600	-0.59367900
H	-1.13928300	-3.65849500	0.84636400
C	0.69426600	-3.57662100	-0.26586700
H	2.56443200	-3.23053200	-1.28895600
H	0.56127400	-4.55221300	-0.71528400
C	-3.48147900	-0.91898800	-0.97946800
C	-2.63257700	-1.76450200	-1.62414200
C	-2.72720500	0.26291100	-0.69721700
H	-2.81455800	-2.76220100	-1.99095800
N	-1.40230700	-1.16967800	-1.79716500
C	-1.42860200	0.06306700	-1.20707900
C	-3.02269000	1.45136500	-0.03312400
H	-0.56346600	-1.65874300	-2.05824100
C	-0.42164900	1.00547500	-1.05942200
C	-2.02927000	2.39158800	0.13846100
H	0.57905000	0.84268100	-1.43985300
C	-0.75019400	2.14808500	-0.36781300
C	-4.90431800	-1.14839500	-0.62748100
H	-5.07068100	-1.04037700	0.44845300
H	-5.56276400	-0.42927600	-1.12141600
H	-5.22985300	-2.14809600	-0.91492100
N	1.06502400	0.07519700	1.55999300
H	1.69993200	0.84449000	1.43105500
C	-0.83559400	-1.01427000	2.06011400
C	-2.17556300	-1.35104000	2.59917100
H	-2.88023800	-1.54674200	1.78431200
H	-2.15112900	-2.24498600	3.22744700
H	-2.57736300	-0.53242200	3.19677400
H	-4.01692600	1.62990500	0.35956000
H	-2.22452800	3.31540100	0.66560800
Br	0.62153500	3.42202200	-0.02935100
Br	3.56278500	-0.55154900	-0.45773600

Entry: 7F-7F
E^{CP} (a.u.): -1004.42507

C	-0.07664000	-1.89303600	1.28767900
C	0.08257300	-0.88753100	2.19048400
C	-1.15980300	-1.50417300	0.43690400
H	0.81171800	-0.79278300	2.97898100
N	-0.84579700	0.10489800	1.97026600
C	-1.60811800	-0.25225000	0.89760900
C	-1.75737400	-2.07770300	-0.68792100
H	-0.89939900	0.98451300	2.44844000
C	-2.63515600	0.41028900	0.25028800
C	-2.77762400	-1.39816200	-1.31816800
H	-1.42166300	-3.03926200	-1.05657000
C	-3.22365000	-0.15235100	-0.85257900
H	-3.25774900	-1.83067000	-2.18639700
H	-4.02663900	0.37710500	-1.34769000
C	0.72027100	-3.13865800	1.17121900
H	0.10973400	-4.02809900	1.34706200
H	1.15247200	-3.23914200	0.17142300
H	1.54158200	-3.14911400	1.88842900
C	0.49154400	2.42629600	-0.31721000
C	-0.33660000	1.95468900	-1.28777300
C	1.52376900	1.44725100	-0.15410200
H	-1.24154100	2.39259400	-1.67921400
N	0.12091300	0.74862400	-1.76903000
C	1.24133400	0.41123300	-1.06172100
C	2.63707100	1.34658300	0.68327100
H	-0.43457500	0.09435200	-2.29434800
C	2.04296100	-0.71472000	-1.11148200
C	3.42722700	0.22042600	0.60676400
C	3.13351200	-0.81710800	-0.28763400

C	-1.43957300	-1.10886700	2.09768300
C	-2.82720000	-1.27359900	2.59379700
H	-3.52313300	-1.39203300	1.75722000
H	-2.93291800	-2.15635200	3.22942900
H	-3.14544200	-0.40489200	3.17052700
H	-4.19914600	1.87611900	0.18259700
H	-2.25316400	3.35625300	0.54137000
I	3.23165200	-1.11558100	-0.35453400
I	0.78503800	3.25539800	0.02878900

Entry: 7Cl-7Cl
E^{CP} (a.u.): -1725.14922

C	0.50716100	-1.42975100	1.77115300
C	0.04197600	-0.33445300	2.42905100
C	-0.44407500	-1.71296300	0.74062600
H	0.48682600	0.20373500	3.25068200
N	-1.15399400	0.07900900	1.88476800
C	-1.46319300	-0.74330300	0.84472300
C	-0.50603600	-2.66588600	-0.27518500
H	-1.67513700	0.89642100	2.14341400
C	-2.52559800	-0.73126500	-0.04339700
C	-1.56781800	-2.63556700	-1.15484800
H	0.27372400	-3.41148700	-0.37343300
C	-2.57964500	-1.67387700	-1.04372100
H	-1.63597800	-3.37164600	-1.94525400
H	-3.41243000	-1.66733600	-1.73413400
C	1.76038400	-2.17723400	2.03750300
H	1.56128400	-3.20681000	2.34579300
H	2.38907500	-2.22506000	1.14340600
H	2.34251300	-1.69744300	2.82485300
C	-0.41447400	2.37272700	-0.63001400
C	-0.80082100	1.48810500	-1.58827000
C	0.88438700	1.95597700	-0.19713700
H	-1.72951400	1.44310400	-2.13533900
N	0.18531300	0.55294200	-1.80311000
C	1.21294700	0.80669900	-0.94391600
C	1.77851800	2.42917000	0.76163200
H	0.08388200	-0.29221200	-2.33746100
C	2.40354000	0.13544300	-0.72218000
C	2.96378300	1.75406200	0.96244600
C	3.28042800	0.60762800	0.22649200
H	3.67141300	2.11068400	1.69939700
H	4.20944900	0.08031700	0.39789200
C	-1.18250800	3.53032800	-0.11050800
H	-1.27658100	3.48861600	0.97922400
H	-0.69966700	4.48207100	-0.34674300
H	-2.18990300	3.55135100	-0.52715800
H	1.54403300	3.31618800	1.33851100
Cl	2.74615000	-1.31309500	-1.63302400
Cl	-3.76120600	0.49078900	0.12232100

Entry: 7I-7I
E^{CP} (a.u.): -1396.27979

C	0.57294900	-0.67723500	2.32371200
C	-0.22802900	0.37887400	2.62534000
C	-0.09833500	-1.40613200	1.29128400
H	-0.06768900	1.16931800	3.34084000
N	-1.37040300	0.34754600	1.85690500
C	-1.31228300	-0.73240400	1.02717800
C	0.22655500	-2.54552600	0.55877300
H	-2.09487500	1.04267000	1.84375100
C	-2.19507600	-1.20082700	0.06474900
C	-0.64932200	-2.99114900	-0.40837300
H	1.15675200	-3.06817100	0.74786300
C	-1.85772500	-2.32848600	-0.65430300
H	-0.41485000	-3.87675900	-0.98474300
H	-2.53699400	-2.71065800	-1.40525500
C	1.87477100	-1.02506100	2.94385500
H	1.82337700	-1.97538000	3.48185800
H	2.66311800	-1.12766800	2.19272600

H	4.29547000	0.12627000	1.24567400
H	3.74838800	-1.70574500	-0.33947800
C	0.35785800	3.68976600	0.44786100
H	0.35228000	3.50156600	1.52611600
H	1.18742100	4.37522400	0.25582600
H	-0.56846200	4.20784800	0.19816600
F	1.72334200	-1.70133400	-1.95707500
H	2.87493900	2.14218400	1.37930500
F	-3.01764600	1.61060600	0.70092900

Entry: 7Br-7Br
E^{CP} (a.u.): -5952.96273

C	0.53228100	-0.92894100	2.13416700
C	-0.18861000	0.14386400	2.55621600
C	-0.22264300	-1.52815700	1.07611200
H	0.05020400	0.86327900	3.32295500
N	-1.35914900	0.24669500	1.83712700
C	-1.39690000	-0.76043200	0.92089900
C	-0.00338000	-2.62024100	0.23829400
H	-2.03391100	0.98699700	1.90141200
C	-2.33753400	-1.08782100	-0.04197200
C	-0.94540600	-2.92819100	-0.72103100
H	0.89515500	-3.21623300	0.34104500
C	-2.11338800	-2.16940300	-0.86278500
H	-0.79436000	-3.77717600	-1.37485000
H	-2.84794700	-2.43053200	-1.61278700
C	1.84330500	-1.39537600	2.64746000
H	1.77453700	-2.39532100	3.08344700
H	2.58737300	-1.44375700	1.84719000
H	2.22662900	-0.72121600	3.41393800
C	-0.62690500	2.21250400	-0.90208000
C	-0.70526100	1.14755500	-1.74465900
C	0.65309400	2.13116500	-0.26870700
H	-1.52030900	0.84535500	-2.38320500
N	0.45650300	0.41227700	-1.69461900
C	1.29279100	0.98371800	-0.78304800
C	1.29905700	2.90103800	0.69655500
H	0.60379100	-0.48641400	-2.11918700
C	2.54989200	0.61193100	-0.33536400
C	2.54882000	2.51620900	1.13321400
C	3.17703200	1.37511300	0.62286300
H	3.06580600	3.10289600	1.88126800
H	4.15763300	1.08608100	0.97728000
C	-1.66383900	3.24342600	-0.65346600
H	-1.94132000	3.28224000	0.40532300
H	-1.31869600	4.24459700	-0.92397100
H	-2.57063400	3.03299200	-1.22101800
H	0.82404500	3.78897700	1.09745400
Br	3.36264900	-0.94829500	-1.04063700
Br	-3.90533200	-0.03637200	-0.21393000

Entry: 4I-7Cl
E^{CP} (a.u.): -1560.71086

C	0.44849200	0.48238300	-1.94247400
C	2.37051100	-0.00037300	-0.93694400
H	-0.46617500	0.27068400	-2.47388600
C	2.14453300	1.38545000	-0.80491900
C	3.49108700	-0.59062700	-0.37497300
C	3.05820700	2.16727600	-0.10024200
C	4.38081800	0.18551300	0.33325900
H	2.89780200	3.23399800	0.00374500
C	4.15971300	1.56192300	0.46667000
H	5.25707600	-0.27701200	0.76701000
H	4.88068700	2.15321300	1.01593500
C	-0.83083200	1.30468100	1.51371000
C	0.36058900	1.51383600	2.13661800
C	-0.83866000	-0.07807500	1.12430600
H	0.74660400	2.43025500	2.55457900
N	1.09044000	0.35471000	2.19113000
C	0.38644400	-0.63625900	1.56873000

H	2.18975300	-0.25749500	3.65138500
C	-0.70366100	2.25900300	-0.96545600
C	-0.69145000	1.12916400	-1.72334900
C	0.55316300	2.29815700	-0.28380800
H	-1.45907600	0.73530800	-2.37095500
N	0.50497100	0.46688400	-1.57321200
C	1.27813200	1.15508700	-0.68415700
C	1.11620200	3.18108700	0.63432800
H	0.71226300	-0.45388500	-1.92035200
C	2.54625400	0.90214300	-0.18137600
C	2.37252200	2.91400500	1.13542800
C	3.08828300	1.78222800	0.73119400
H	2.82677100	3.58967300	1.84844700
H	4.07542600	1.59700400	1.13474100
C	-1.78925400	3.26174400	-0.84035000
H	-2.12589500	3.35788800	0.19725900
H	-1.46029200	4.25510000	-1.15656300
H	-2.65555500	2.98733100	-1.44271800
H	0.57204700	4.06514500	0.94618800
I	3.60199400	-0.79897800	-0.81572400
I	-4.01271900	-0.20401200	-0.27216900

Entry: 5Cl-6F
E^{CP} (a.u.): -1364.78606

C	0.12587100	1.15819000	-1.90447600
C	1.34847700	-0.53541200	-1.11845200
H	-0.73353000	1.62126800	-2.36317900
C	2.04858100	0.66614000	-0.88481300
C	1.84245800	-1.76955200	-0.71350700
C	3.27545500	0.62324000	-0.22732300
C	3.05282500	-1.75507300	-0.06368800
H	1.32112400	-2.70120300	-0.88859900
H	3.82907800	1.53607600	-0.04102600
C	3.77727500	-0.59183800	0.18934700
H	4.72756300	-0.67388500	0.69897300
C	-0.66571900	1.67875000	1.56758000
C	0.41613400	1.13488000	2.18766700
C	-1.42149300	0.58070400	1.05487600
H	1.23725500	1.63077100	2.68085100
N	0.37097200	-0.23834500	2.11290400
C	-0.74054400	-0.60293300	1.40357100
C	-2.59464200	0.50515100	0.30595100
H	1.11994200	-0.85524100	2.36925900
C	-1.20218500	-1.85384900	1.02095600
C	-3.03281600	-0.74045000	-0.07754200
H	-0.67213600	-2.75785300	1.29390700
C	-2.35682500	-1.91467000	0.26815800
H	-2.75813500	-2.86639100	-0.05186600
C	-0.99556300	3.11408100	1.39807900
H	-1.01806800	3.38682500	0.33779000
H	-1.97739400	3.35744000	1.81169500
H	-0.26067500	3.75269500	1.88898600
N	0.18797900	-0.21311000	-1.76073500
H	-0.57924200	-0.84241700	-1.91737800
C	1.24591300	1.73455400	-1.39693900
C	1.58099000	3.17726700	-1.33231900
H	1.68998500	3.50861000	-0.29438700
H	2.52454400	3.39861500	-1.83759100
H	0.80501200	3.78800700	-1.79499100
H	-3.14298400	1.39469200	0.02181900
Cl	-4.49212100	-0.87714500	-1.03584100
F	3.56843600	-2.91793800	0.35189900

Entry: 6F-6Br
E^{CP} (a.u.): -3478.69367

C	-1.58718000	2.36770900	-1.39506600
C	0.14732600	0.98950800	-1.15587400
H	-2.57737200	2.66034300	-1.70633600
C	0.46970000	2.21740300	-0.54376700
C	1.01099800	-0.09582600	-1.13724800

C	-1.70133000	-0.92900000	0.42599900
H	2.05611700	0.28858700	2.45941100
C	0.74024600	-1.96079800	1.36277800
C	-1.36095300	-2.24832800	0.20951900
H	1.69077500	-2.34386700	1.71303500
C	-0.14804800	-2.76050100	0.68077200
H	-2.03985800	-2.89610100	-0.32911900
H	0.09083600	-3.80071500	0.50040400
C	-1.86673900	2.35189200	1.31238200
H	-2.07533300	2.52523800	0.25423500
H	-2.81659400	2.09146300	1.78292900
H	-1.52915100	3.29612200	1.74397700
N	1.33764800	-0.52787900	-1.65136900
H	1.15951300	-1.51127700	-1.75406700
C	0.90767100	1.66971200	-1.46328600
C	0.26303600	3.00101200	-1.56745500
H	0.10761800	3.44033100	-0.57711700
H	0.87336100	3.70532700	-2.13862000
H	-0.71018600	2.93082600	-2.05537500
Cl	3.75087700	-2.30448200	-0.57632000
I	-3.52213800	-0.25786800	-0.39839000

Entry: 5Cl-7Br
E^{CP} (a.u.): -3839.05363

C	0.25189600	0.88775300	2.15521900
C	-1.43079700	0.44304300	0.77068600
H	1.03833200	0.65540300	2.85546800
C	-1.18803400	1.83286100	0.73583500
C	-2.42244600	-0.12265500	-0.01585500
C	-1.95047700	2.64381000	-0.10230500
C	-3.16009500	0.68513400	-0.85282800
H	-1.77602700	3.71312800	-0.13319000
C	-2.92116400	2.06472300	-0.89223400
H	-3.93612700	0.24781300	-1.46651500
H	-3.52546800	2.67857000	-1.54740700
C	2.37956300	1.79867400	-0.88370600
C	1.33113900	2.24701300	-1.62614600
C	2.19526400	0.38821800	-0.74898200
H	1.09763100	3.25493600	-1.93253700
N	0.51553200	1.19935900	-1.98627900
C	1.01358100	0.04848000	-1.43719100
C	2.91427200	-0.60350600	-0.08344300
H	-0.40420800	1.29690500	-2.37866500
C	0.53475700	-1.25250000	-1.47350300
C	2.42191000	-1.88679400	-0.12173100
H	-0.38016100	-1.50453400	-1.99559600
C	1.24940000	-2.22276200	-0.80327300
H	0.91110600	-3.24996000	-0.79870400
C	3.48703000	2.58919400	-0.29467800
H	3.51494500	2.47634300	0.79378600
H	4.46125600	2.26698100	-0.67102200
H	3.38072900	3.65184400	-0.51556900
N	-0.55380700	-0.11011800	1.65400500
H	-0.42314400	-1.09704500	1.78913600
C	-0.10716700	2.09182700	1.63437900
C	0.50089200	3.41819100	1.89802800
H	0.94056300	3.83414300	0.98556800
H	-0.23878700	4.13814200	2.25689300
H	1.29086800	3.35212100	2.64644100
H	3.82754000	-0.37843500	0.45287400
Cl	3.28756900	-3.15965300	0.71406600
Br	-2.74277000	-1.98717600	0.07703500

Entry: 4F-6I
E^{CP} (a.u.): -1200.35224

C	-2.50467100	1.70005900	-1.45488800
C	-0.41078500	1.00566600	-1.14159800
H	-3.51395800	1.64803200	-1.83127900
C	-0.59516100	2.19924200	-0.41524200
C	0.77931700	0.29455100	-1.10676600

C	1.69951600	2.35749900	0.09529600
C	2.20073900	0.07985600	-0.47027200
H	0.75357000	-1.04117500	-1.59718200
H	1.97051700	3.29473800	0.56723300
C	2.56295700	1.28394900	0.13808600
H	3.51748700	1.35901600	0.64066500
C	-0.80560600	0.12317500	2.07319900
C	0.25022700	-0.72858500	2.12885200
C	-1.75587000	-0.47524300	1.18558100
H	1.17971400	-0.63080700	2.66696300
N	0.01134400	-1.83692700	1.34117600
C	-1.20184600	-1.69171200	0.73259900
C	-3.00784100	-0.08267900	0.72054300
H	0.69673500	-2.53651000	1.12011300
C	-1.85512600	-2.50495000	-0.18621700
C	-3.67379800	-0.87979600	-0.18787500
H	-1.44851300	-3.44012200	-0.54706700
C	-3.08367000	-2.06593200	-0.62008400
C	-0.95382400	1.43250300	2.75297200
H	-1.10961700	2.23165600	2.02116700
H	-1.81044800	1.44531700	3.43189300
H	-0.06370000	1.68030500	3.33183700
C	-0.65410000	3.08552100	-0.71298100
C	-0.75326800	4.48505200	-0.23094600
H	-0.59731200	4.54595700	0.85013900
H	0.00173600	5.12517100	-0.69450500
H	-1.73113800	4.91367200	-0.44994800
N	-1.10232200	1.11390000	-1.69478400
H	-1.66247700	0.34752400	-2.02426100
H	-3.45080300	0.84531100	1.06261800
H	-4.65180300	-0.61891200	-0.56894300
Br	3.38149300	-1.40462200	-0.32120900
F	-3.75422200	-2.81591600	-1.50084500

C	0.45347300	2.69439500	0.35629800
C	1.78117400	0.80296600	-0.30910700
H	0.90053900	-0.63018700	-1.65708300
H	0.33626400	3.61344100	0.91883200
C	1.63854800	1.99171500	0.41452300
H	2.45849000	2.35122600	1.02167700
C	-1.43010900	-0.46830100	1.83169400
C	-0.13961500	-0.86335700	1.99966100
C	-1.95054000	-1.28581400	0.77890900
H	0.60480500	-0.47643100	2.67755300
N	0.16977300	-1.88373100	1.12987300
C	-0.91742100	-2.14275400	0.34417800
C	-3.17039000	-1.37666000	0.11916600
H	1.09598100	-2.24258500	0.97748500
C	-1.07052600	-3.02461400	-0.72109400
C	-3.35341800	-2.23677000	-0.93485300
H	-0.25815000	-3.66461500	-1.04056800
C	-2.29549200	-3.05492100	-1.35239600
H	-4.31993000	-2.27847500	-1.41848000
H	-2.45292100	-3.73193900	-2.18160100
C	-2.12767100	0.62399600	2.55623300
H	-2.45452600	1.40421200	1.86209100
H	-3.01708000	0.26762400	3.07920400
H	-1.46365000	1.08375400	3.28911900
N	-1.58141100	0.73583600	-1.79427900
H	-1.80361800	-0.14176100	-2.23180600
C	-1.94154800	2.62758800	-0.63413100
C	-2.57423800	3.84092800	-0.06100300
H	-2.51842100	3.84060100	1.03157400
H	-2.07880700	4.75373400	-0.40162800
H	-3.62584600	3.90991900	-0.33858800
F	-4.18065900	-0.59575300	0.50926300
I	3.55835200	-0.30711300	-0.10012300

Coordinates of toluene monomers

Electronic energies are provided at M06/cc-PVTZ(-PP) level of theory.

All structures are characterized by 0 imaginary frequencies.

Entry: H

E_{elec} **(a.u.):** -271.42952

E_{elec} **N-1 system (a.u.):** -271.10900

E_{elec} **N+1 system (a.u.):** -271.36727

C	1.88855600	0.00559800	-0.00949400
C	1.19206700	1.19661400	0.09499300
C	-0.19252000	1.19362700	0.10632300
C	-0.90541000	0.00588800	0.01236600
C	-0.19328300	-1.18364100	-0.08826900
C	1.18970100	-1.18680800	-0.10030200
H	2.97078400	0.00514600	-0.01592200
H	-0.73633800	-2.12023800	-0.15518700
H	1.72543200	-2.12450500	-0.17724400
C	-2.40059400	0.00131200	-0.00249300
H	-2.80798500	0.92161900	0.41608500
H	-2.78373800	-0.09143200	-1.02161700
H	-2.80213600	-0.83790800	0.56711000
H	1.72869200	2.13383700	0.17128200
H	-0.73342900	2.12964700	0.19203500

Entry: *o*F

E_{elec} **(a.u.):** -370.67597

E_{elec} **N-1 system (a.u.):** -370.35247

E_{elec} **N+1 system (a.u.):** -370.61965

C	1.88771400	0.00368400	-0.00109800
C	1.18836900	1.19763800	0.04885000
C	-0.18787500	1.16008200	0.04894600
C	-0.91459600	-0.01889200	0.00132700
C	-0.18516600	-1.19846100	-0.04805700
C	1.19986400	-1.19556800	-0.04960000
H	2.96952500	0.01464100	-0.00195400
H	-0.72400500	-2.13833700	-0.08598200
H	1.74096700	-2.13152200	-0.08860900
C	-2.40627500	0.00869500	0.00407600
H	-2.79305600	0.49581100	0.90096700
H	-2.79470700	0.57120500	-0.84680500
H	-2.81304600	-1.00055100	-0.03901700
H	1.68969900	2.15526800	0.08746800
F	-0.86071500	2.31452400	0.09702800

Entry: *m*F

E_{elec} **(a.u.):** -370.67495

E_{elec} **N-1 system (a.u.):** -370.34979

E_{elec} **N+1 system (a.u.):** -370.62373

C	1.89518100	-0.00117300	-0.00949400
C	1.17296400	1.17152600	0.07110400
C	-0.20368400	1.18873500	0.08291600
C	-0.90526100	-0.00886400	0.01077000
C	-0.19037300	-1.19638800	-0.06889200
C	1.19471800	-1.19124600	-0.07940900
H	2.97572900	0.03403600	-0.01344500
H	-0.72760300	-2.13575400	-0.12151200
H	1.73623100	-2.12627000	-0.14053600

Entry: *p*F

E_{elec} **(a.u.):** -370.67415

E_{elec} **N-1 system (a.u.):** -370.35515

E_{elec} **N+1 system (a.u.):** -370.62265

C	1.86287300	0.00022400	-0.00597800
C	1.19477300	1.20063800	0.09363300
C	-0.18942900	1.19130300	0.10323700
C	-0.90594200	0.00532600	0.01383400
C	-0.19422400	-1.18487300	-0.08119600
C	1.18839800	-1.19917500	-0.09279500
H	-0.73593400	-2.12190900	-0.14472500
H	1.74875900	-2.12155100	-0.16365600
C	-2.40150000	0.00386700	-0.00255800

C	-2.39993700	0.00036100	0.00548900
H	-2.79558000	0.63094500	0.80319000
H	-2.78618500	0.39751300	-0.93579700
H	-2.80593400	-1.00248300	0.13289300
F	1.83006900	2.32933200	0.14221700
H	-0.72127900	2.13816300	0.15005300

Entry: *o*Cl
E_{elec} (a.u.): -731.03556
E_{elec} N-1 system (a.u.): -730.71464
E_{elec} N+1 system (a.u.): -730.98435

C	1.88588700	0.00231600	-0.00244200
C	1.19238200	1.19752700	0.04478600
C	-0.18945200	1.18046200	0.04574100
C	-0.92050400	-0.00132900	0.00122800
C	-0.19302800	-1.18503400	-0.04561400
C	1.18984000	-1.19245600	-0.04796200
H	-0.73725900	-2.12174500	-0.08097400
H	1.72379100	-2.13277000	-0.08519000
C	-2.41114500	-0.01105400	0.00287700
H	-2.80996800	0.47638700	0.89458100
H	-2.81203000	0.53476700	-0.85342400
H	-2.79175500	-1.03080600	-0.03097700
H	2.96768400	0.00892300	-0.00357800
H	1.71124000	2.14566600	0.08071700
Cl	-1.03314000	2.71085700	0.10445600

Entry: *p*Cl
E_{elec} (a.u.): -731.03386
E_{elec} N-1 system (a.u.): -730.71829
E_{elec} N+1 system (a.u.): -730.98806

C	1.87187300	-0.00089400	-0.00226500
C	1.19662000	1.19864300	0.10442400
C	-0.18727500	1.19248000	0.11149000
C	-0.90544800	0.00864500	0.01270700
C	-0.19539900	-1.18188900	-0.08946100
C	1.18659900	-1.19748000	-0.09866600
H	-0.73720700	-2.11839200	-0.16075300
H	1.73459800	-2.12658600	-0.17621500
C	-2.40013900	0.00829200	-0.00495800
H	-2.80523900	0.93550200	0.39971200
H	-2.78190200	-0.09852100	-1.02306200
H	-2.80487400	-0.82095600	0.57662000
H	-0.72093900	2.13204100	0.19823800
H	1.75117700	2.12368600	0.18377000
Cl	3.61629600	-0.00812100	-0.00920500

Entry: *m*Br
E_{elec} (a.u.): -2844.94036
E_{elec} N-1 system (a.u.): -2844.62427
E_{elec} N+1 system (a.u.): -2844.89710

C	1.89501800	0.00022000	-0.02927500
C	1.18874100	1.18394800	0.05717100
C	-0.19356100	1.20155600	0.07894500
C	-0.90408500	0.01090300	0.01159000
C	-0.19632500	-1.18246600	-0.07210100
C	1.18579500	-1.18758000	-0.09329300
H	-0.74138500	-2.11831900	-0.11827600
H	1.72260200	-2.12531900	-0.15680500
C	-2.39895300	0.01324500	0.00779600
H	-2.79986600	0.94779300	0.39901100
H	-2.78721800	-0.11166800	-1.00560200
H	-2.79764300	-0.80619100	0.60693600
H	2.97609300	0.00863000	-0.04198800
Br	2.13412400	2.82524300	0.15406400
H	-0.72005200	2.14546800	0.15083500

Entry: *o*I

H	-2.80648800	0.92123100	0.42457700
H	-2.78676700	-0.07867600	-1.02168600
H	-2.80552700	-0.83788800	0.56131100
H	-0.72592700	2.12969800	0.18461600
H	1.75902200	2.12053600	0.16548900
F	3.19665900	-0.00323700	-0.01328900

Entry: *m*Cl
E_{elec} (a.u.): -731.03418
E_{elec} N-1 system (a.u.): -730.71309
E_{elec} N+1 system (a.u.): -730.98729

C	1.89440000	-0.00007400	-0.02665500
C	1.18296500	1.18260200	0.03655000
C	-0.19785100	1.19761300	0.05772600
C	-0.90474100	0.00299200	0.01248700
C	-0.19403100	-1.18899900	-0.04758100
C	1.18932700	-1.18974400	-0.06779700
H	-0.73548600	-2.12735500	-0.07577800
H	1.72876700	-2.12697000	-0.11244400
C	-2.39956200	0.01004000	0.00340000
H	-2.79882100	0.85030000	0.57159900
H	-2.78336000	0.09745900	-1.01573400
H	-2.80421200	-0.91027000	0.42442700
H	-0.72253800	2.14365400	0.11195000
Cl	2.04921500	2.69599400	0.09545000
H	2.97518900	0.01776100	-0.03846200

Entry: *o*Br
E_{elec} (a.u.): -2844.94150
E_{elec} N-1 system (a.u.): -2844.62482
E_{elec} N+1 system (a.u.): -2844.89680

C	1.88522300	0.00275300	-0.00292300
C	1.19244200	1.19873500	0.04144700
C	-0.19034300	1.18649200	0.04290400
C	-0.92211500	0.00401800	0.00083700
C	-0.19512200	-1.18097100	-0.04314800
C	1.18742500	-1.19076200	-0.04528400
H	-0.74021000	-2.11730300	-0.07629400
H	1.71963200	-2.13222400	-0.08001300
C	-2.41325700	-0.01788000	0.00196000
H	-2.81694400	0.45907400	0.89728400
H	-2.81919100	0.52946200	-0.85104800
H	-2.78501100	-1.04074500	-0.03854600
H	2.96711300	0.00854000	-0.00418800
H	1.71858400	2.14315900	0.07490000
Br	-1.09094500	2.85911000	0.10513700

Entry: *p*Br
E_{elec} (a.u.): -2844.94014
E_{elec} N-1 system (a.u.): -2844.62902
E_{elec} N+1 system (a.u.): -2844.89578

C	1.87391600	0.00009300	-0.00093800
C	1.19358800	1.20062700	0.07713400
C	-0.18960300	1.19146900	0.08474000
C	-0.90520000	0.00300600	0.01357400
C	-0.19301400	-1.18711300	-0.05985200
C	1.19035900	-1.19905500	-0.06832700
H	-0.73193200	-2.12661800	-0.10832100
H	1.73533000	-2.13176800	-0.12276500
C	-2.39967800	0.00730400	-0.00800400
H	-2.80449400	0.84888600	0.55450500
H	-2.77754800	0.09038700	-1.02988900
H	-2.80786900	-0.91145800	0.41341700
H	-0.72602200	2.13141100	0.15026600
H	1.74114400	2.13164000	0.13470800
Br	3.76976400	-0.00201800	-0.00730400

Entry: *m*I

E_{elec} (a.u.): -566.59878
E_{elec} N-1 system (a.u.): -566.29324
E_{elec} N+1 system (a.u.): -566.55072

C	1.88790400	0.00243300	0.00738100
C	1.18731300	1.19720500	0.00175600
C	-0.19559900	1.19125000	-0.00746500
C	-0.90608400	-0.00337700	-0.00946500
C	-0.19141700	-1.19378300	-0.00747100
C	1.19286400	-1.19409600	0.00178900
H	-0.73142600	-2.13426300	-0.01623500
H	1.73066000	-2.13381300	0.00100500
C	-2.40094000	-0.00138300	0.00754100
H	-2.78323100	0.15479300	1.01923600
H	-2.80596700	0.79833400	-0.61398500
H	-2.80610700	-0.94790900	-0.35057900
H	2.95789346	0.00487532	0.01145482
H	1.71535428	2.12783463	0.00081228
I	-1.24926939	3.00770156	-0.02427126

E_{elec} (a.u.): -566.59796
E_{elec} N-1 system (a.u.): -566.29350
E_{elec} N+1 system (a.u.): -566.55989

C	1.89205700	0.00075500	0.01210900
C	1.18441300	1.18881900	-0.02994500
C	-0.20001400	1.19615800	-0.04243700
C	-0.90644300	0.00078200	-0.01018300
C	-0.19405900	-1.19167200	0.02866500
C	1.18817700	-1.19176100	0.04049200
H	-0.73517500	-2.13082700	0.04706300
H	1.72924500	-2.12895000	0.06883100
C	-2.40129600	-0.00127700	0.00543300
H	-2.78117000	-0.12169700	1.02261900
H	-2.80780700	0.93062900	-0.38658000
H	-2.80267600	-0.82440800	-0.58665900
H	-0.73908900	2.13557600	-0.07948500
I	2.22720800	3.01477800	-0.07970200
H	2.97367700	0.00381300	0.01861800

Entry: *p*I
E_{elec} (a.u.): -566.59784
E_{elec} N-1 system (a.u.): -566.29767
E_{elec} N+1 system (a.u.): -566.55496

C	1.87925800	-0.00286600	0.01461700
C	1.19276900	1.18049100	0.22642900
C	-0.19067900	1.17537100	0.20929500
C	-0.90630600	0.00624200	-0.01455900
C	-0.19384900	-1.16651300	-0.22842600
C	1.18971300	-1.18098200	-0.21492600
H	-0.73244200	-2.08940700	-0.41278700
H	1.72699400	-2.10455400	-0.38590700
C	-2.40072500	0.00634400	-0.00136300
H	-2.80282500	0.96167100	-0.33928400
H	-2.80579700	-0.77876200	-0.64003700
H	-2.78423300	-0.16689600	1.00704200
H	-0.72693100	2.10387500	0.37107200
H	1.73252700	2.10208200	0.40029800
I	3.98126200	-0.00887000	0.03220300
